# The Phytocyanin Gene Family in Rice (*Oryza sativa* L.): Genome-Wide Identification, Classification and Transcriptional Analysis

**DOI:** 10.1371/journal.pone.0025184

**Published:** 2011-10-03

**Authors:** Haoli Ma, Heming Zhao, Zhi Liu, Jie Zhao

**Affiliations:** State Key Laboratory of Hybrid Rice, College of Life Sciences, Wuhan University, Wuhan, China; Purdue University, United States of America

## Abstract

**Background:**

Phytocyanins (PCs) are plant-specific blue copper proteins involved in electron transport, and a large number of known PCs are considered to be chimeric arabinogalactan proteins (AGPs). To date there has not been a genome-wide overview of the *OsPC* gene family. Therefore, as the first step and a useful strategy to elucidate the functions of OsPCs, there is an urgent need for a thorough genome-wide analysis of this gene family.

**Methodology/Principal Findings:**

In this study, a total of 62 *OsPC* genes were identified through a comprehensive bioinformatics analysis of the rice (*Oryza sativa* L.) genome. Based on phylogeny and motif constitution, the family of OsPCs was classified into three subclasses: uclacyanin-like proteins (OsUCLs), stellacyanin-like proteins (OsSCLs) and early nodulin-like proteins (OsENODLs). Structure and glycosylation prediction indicated that 46 OsPCs were glycosylphosphatigylinositol-anchored proteins and 38 OsPCs were chimeric AGPs. Gene duplication analysis revealed that chromosomal segment and tandem duplications contributed almost equally to the expansion of this gene family, and duplication events were mostly happened in the OsUCL subfamily. The expression profiles of *OsPC* genes were analyzed at different stages of vegetative and reproductive development and under abiotic stresses. It revealed that a large number of *OsPC* genes were abundantly expressed in the various stages of development. Moreover, 17 genes were regulated under the treatments of abiotic stresses.

**Conclusions/Significance:**

The genome-wide identification and expression analysis of *OsPC* genes should facilitate research in this gene family and give new insights toward elucidating their functions in higher plants.

## Introduction

Phytocyanins (PCs) are ancient blue copper proteins which can bind with a single copper atom and function as electron transporter, and uclacyanins (UCs) and stellacyanins (SCs) are typical family members of PCs [1]–[5]. Early nodulin (ENOD)-like proteins (ENODLs) have structures similar to UCs and SCs but lack key amino acid residues responsible for copper binding, and are considered to be involved in processes without binding with copper [6], [7]. Two ENODLs were identified as proteins reactive to β-glucosyl Yariv reagent, a synthetic phenylglycoside dye that binds specifically with arabinogalactan proteins (AGPs), from *Arabidopsis thaliana* and rice (*Oryza sativa* L.), respectively [7], [8]. It was reported that 25 and 36 plastocyanin-like domain (PCLD)-containing proteins (UC-like, SC-like and ENOD-like) with a glycosylphosphatidylinisotol-anchor signal (GAS) were identified in *Arabidopsis* and rice, respectively, and 40% of them had putative arabinogalactan (AG) glycomodules [9], [10]. Due to the presence of AGP-like regions in most PCs, the *PC* gene family is usually classified as a subfamily of the AGP superfamily.

AGPs are a highly diverse class of cell surface glycoproteins that consist of a core protein backbone O-glycosylated by large type II AG polysaccharide chains, which comprise more than 90% of their molecular weight [11]. A bioinformatics approach for searching hydroxyproline-rich glycoproteins identified 17 PC-like AGPs in *Arabidopsis* [12]. In addition, three ENOD-like AGPs were identified in rice by calculating the proportion of proline, alanine, serine and threonine (PAST), as >35% PAST is an important characteristic of AGPs [13]. Recently, a total of 38 PCs were identified and classified into several groups based on their domain composition and phylogenetic relationship in *Arabidopsis*, including eight AtUCs, four AtSCs, one plantacyanin, 22 AtENODLs, and three unknown PCLD-containing proteins, 18 of which were regarded as chimeric AGPs [14].

The biochemical characteristics of the spectroscopic and redox properties of PCs have been reported in many previous studies, but there are few reports describing their functions in the different developmental processes [2]–[5]. Chemocyanin from lily stigma which was a member of *PC* gene family and induced pollen tube chemotropism was isolated by using a serial of biochemical methods [3]. Overexpression of *Arabidopsis* plantacyanin reduced the seed set and inhibited the germination of pollen grains, and the guidance of wild-type pollen tubes on the overexpression stigma was also disrupted [4]. Several *ENODL* genes were found specifically expressed in apical buds in *Pharbitis nil*, indicating that ENODLs may function not only in the processes of nodulation but also in the organ development [15]. Recently, it was reported that a sieve element-specific ENODL was involved in the determination of reproductive potential in *Arabidopsis* [16].

In this study, we firstly identified genes coding for OsPCs and divided them into three subfamilies. We further analyzed the amino acid constitution and protein modifications of OsPCs, and conducted a phylogenetic analysis of OsPCs and AtPCs. Moreover, we evaluated publicly available high-throughput transcriptional analyses to select specific experiments for interesting genes, such as massively parallel signature sequencing (MPSS) and microarrays. The data from the digital expression analysis were validated by quantitative real-time RT-PCR (qRT-PCR). Our results indicate important physiological functions of OsPCs and are a solid base for research on the functions of the *OsPC* gene family.

## Results

### Identification and classification of OsPCs

BLASTP searches using PCLDs of 26 known PCs and name searches using multiple keywords were used to obtain the amino acid sequences of PCs against several rice protein databases, and to uncover the entire family of genes coding for PCs in the rice genome. After confirming the presence of PCLDs and removing the redundancies, we identified a total of 62 OsPCs in the rice genome (Table S1).

**Figure 1:**
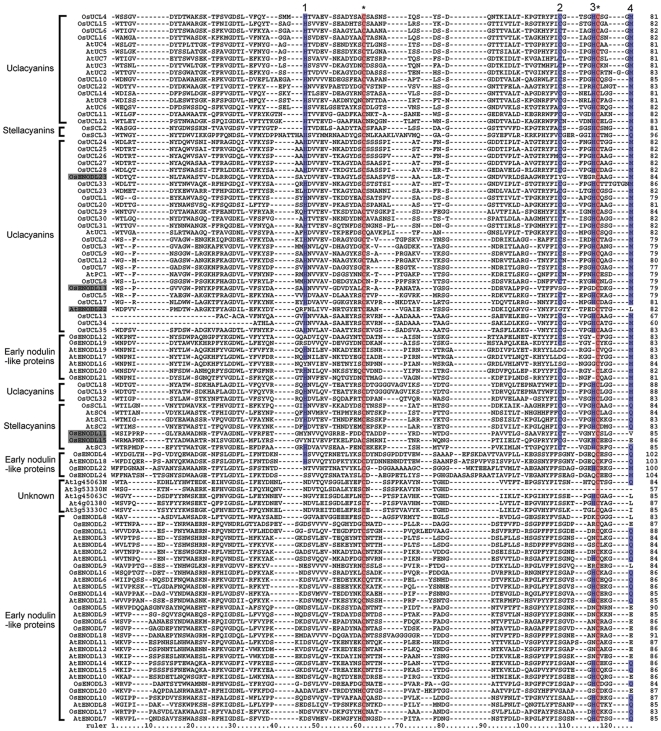
Multiple sequence alignment of the plastocyanin-like domains (PCLDs) of rice and *Arabidopsis* PCs. The conversed amino acids involved in copper binding are marked on blue background (His, Cys, His, and Gln/Met), while the Cys residues involved in the disulfide linkage are indicated on red background. The ENODLs exist in clades of UCs and SCs are shown on gray background.

A multiple sequence alignment was conducted using the PCLDs of 62 OsPCs and 38 AtPCs, and so to clarify the sequence characteristics of OsPCs [14] (Figure 1). It was noteworthy that the Cys residues involved in the formation of disulfide linkages were highly conserved in both the OsPCs and AtPCs, implying that the disulfide linkage was important for stability of PCLD structure. The 38 OsPCs with four amino acid residues (His, Cys, His, and Met/Gln) involved in copper binding were divided into two types: 35 OsUCLs and three OsSCLs. The remaining 24 OsPCs that had a PCLD lacking key residues for copper binding were defined as OsENODLs. In previous studies, OsENODL1 was identified by isolating β-glucosyl Yariv reactive proteins [7] and OsENODL1, OsENODL6 and OsENODL18 were named OsELA1, OsELA2 and OsELA3, respectively [13]. To sum up, 62 OsPCs were categorized into OsUCLs, OsSCLs and OsENODLs and named in accordance with chromosome order (Table S1).

### Structure and phylogenetic analysis

In order to investigate the characteristics of OsPCs, the predictions of signal peptides (SPs), AG glycomodules, N-glycosylation sites and GASs were determined using several bioinformatics websites (Tables S1 and S2). Sixty OsPCs were expected to have an N-terminal secretion signal responsible for targeting to the endoplasmic reticulum. The majority of OsPCs (46 of 62) were found to be GPI-anchored proteins, indicating that these proteins might localize in the plasma membrane. Moreover, 38 OsPCs had putative AG glycomodules in the PAST-rich region, all of which were predicted to be GPI-anchored proteins. In addition, most of these proteins had putative N-glycosylation sites in the PCLD and PAST-rich region. The existence of SPs and AG glycomodules indicated that these 38 OsPCs might be chimeric AGPs.

**Figure 2:**
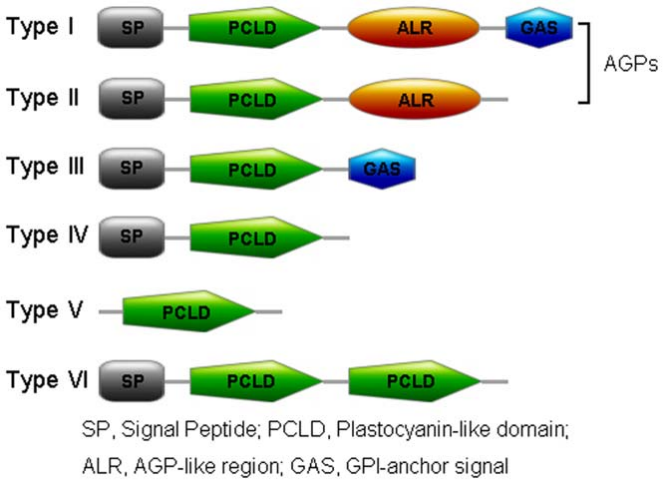
Schematic representations of six types of rice and *Arabidopsis* PCs. Not drawn to scale. The domain features of PCs are generated by MyDomains (http://www.expasy.ch/cgi-bin/prosite/mydomains/).

Due to the presence of the N-terminal SP, AG glycomodules and C-terminal GAS, PCs were divided into six types (Figure 2). Type I PCs had typical properties of PCs, including an N-terminal SP, a PCLD, an AGP-like region (ALR) and a C-terminal GAS. Types II and III PCs were similar to type I PCs but lack GAS and ALR, respectively. Both ALR and GAS were absent from type IV PCs. Type V PCs only had a PCLD; and type VI PCs had SP and two PCLDs but lack ALR and GAS. Moreover, types I and II PCs were considered to be chimeric AGPs, as the existence of N-terminal SP and AG glycomodules was sufficient for glycosylating by a large branched AG polysaccharide. There were 38 type I OsPCs (19 OsUCLs, OsSCL1 and 18 OsENODLs); eight type III OsPCs (OsUCL15, OsUCL18, OsUCL31, OsSCL2, OsENODL12, OsENODL15, OsENODL19 and OsENODL21); 14 type IV OsPCs (11 OsUCLs, OsSCL3, OsENODL11 and OsENOD13); and two type V OsPCs (OsUCL13 and OsUCL34) (Tables 1 and S2). To conclude, there were 38 PC-like AGPs in rice (type I and type II), including 19 OsUCLs, OsSCL1 and 18 OsENODLs.

**Table 1:** Classifications of rice and *Arabidopsis* PCs.

Type	OsUCLs	OsSCLs	OsENODLs	AtUCs	AtSCs	AtENODLs	AtUnkown
I	19	1	18^a^	7	4	16	-
II	-	-	-	-	-	2	-
III	3	1	4	1	-	3	-
IV	11	1	2	1^b^	-	1	-
V	2	-	-	-	-	-	1
VI	-	-	-	-	-	-	2

a , OsENODL14 with several A/TP_2–3_ motifs is identified as a putative AGPs in present study.

b , The *Arabidopsis* plantacyanin is recognized as a member of UCs here.

“-” means not exist.

**Figure 3:**
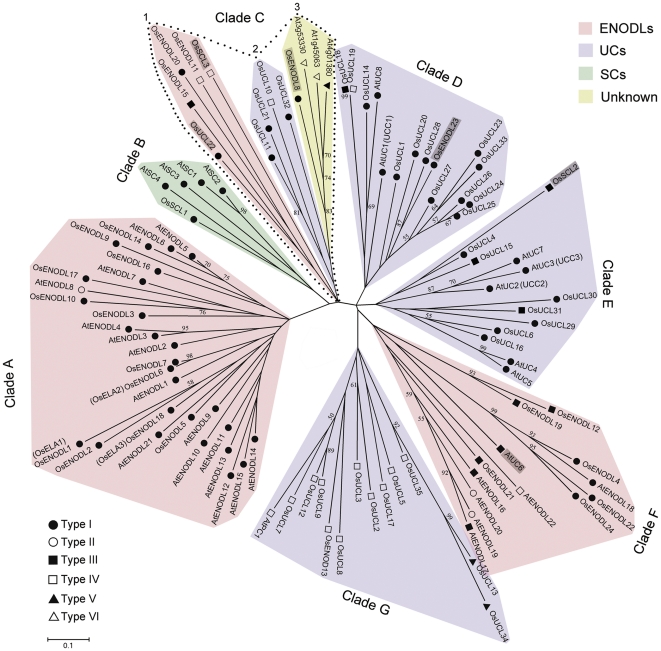
Phylogenetic analysis of rice and *Arabidopsis* PCs. Six types of PCs are marked by solid round (type I), round (type II), solid square (type III), square (type IV), solid triangle (type V), triangle (type VI). The ENODLs exist in clades of UCs and SCs are shown on a gray background. Scale bar represents 0.1 amino acid substitution per site.

An unrooted phylogenetic tree was generated from alignments of full-length protein sequences among OsPCs and AtPCs to analyze their evolutionary relationships (Figure 3). Based on their sequence homology, with few exceptions, the entire PCs were clustered into distinct clades representing different subfamilies. Manual analysis of the phylogenetic tree revealed seven distinct clades (A–G) of OsPCs and AtPCs. Therefore, most members of each subfamily were clustered together. For instance, most PCs in clades A and F belonged to the ENODL subfamily, and all ENODLs in clade A were ENOD-like AGPs. Five members of the SCL subfamily were grouped in clade B, while the two members of OsSCLs were phylogenetically close to UCs. Similarly to ENODLs, clades D, E and G mostly contained members of the UC subfamily. Most UCs in clades D and E were UC-like AGPs, but all UCs in clade G lacked both the AG glycomodules and GASs. Clade C contained three subclades: mainly ENODLs and UCs in subclades 1 and 2, respectively; and three PCLD-containing proteins classified as unknown in subclade 3. Additionally, the phylogeny of OsPCs and AtPCs was determined using the Bayesian estimation of MrBayes program (Figure S1). There were similar results for the Neighbor Joining and Bayesian estimation methods. The majority of UCs was grouped into two clades and most ENODLs were clustered in three clades. The three AtPCs of unknown type (At4g01380, At1g45063 and At3g53330) were phylogenetically close to the members of SCs. Moreover, the major difference was that OsENODL15, OsENODL18, AtUC6 and AtUC8 had low similarity to other PCs in the Bayesian estimation. The two methods of phylogeny analysis showed that several clades contained representatives from both rice and *Arabidopsis*, implying that there was a common ancestor of each subfamily before the divergence of monocot and dicot lineages. Nevertheless, within a clade, there was also species-specific clustering of PCs, indicating that expansion of the PC subfamilies occurred independently in rice and *Arabidopsis* after their divergence.

### Chromosomal localization and gene duplication

**Figure 4:**
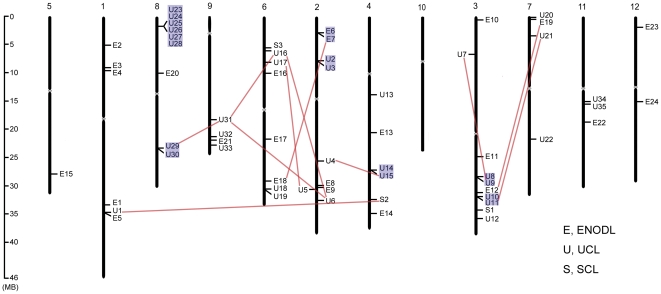
Genomic localization of *PC* genes in rice chromosomes. White ovals on the chromosomes indicate the position of centromeres. Chromosome numbers are indicated at the top of each chromosome. Genes present on duplicated segments of genome are connected by red lines, and tandem duplicated genes are marked on a blue background.

The exact coordinates and orientation of *OsPC* genes in the rice chromosomes are available in the Rice Genome Annotation Project (RGAP) database; and the locations of *OsPC* genes are approximately marked in the rice chromosome based on the pseudomolecules at which their open reading frames locate (Figure 4). The 62 *OsPC* genes were randomly distributed in 11 rice chromosomes, and there was no substantial clustering of *OsPC* genes in rice chromosomes. There were ten genes present in chromosome 3, followed by nine each in chromosomes 2 and 8, eight genes in chromosome 6, six each in chromosomes 1 and 4, four each in chromosomes 7 and 9; three in chromosome 11, two in chromosomes 12, and only one in chromosome 5.

Both segmental and tandem gene duplications had great impact on the expansion and evolution of gene families in plant genomes. The large number of members of the *OsPC* gene family indicated that it evolved through a large number of duplication events. Therefore, we investigated the contributions of segmental and tandem duplications on the expansion of the *OsPC* gene family. Twenty *OsPC* genes were found to be located in the duplicated chromosomal segments of rice chromosomes mapped by RGAP using a maximal distance between collinear gene pairs of 500 kb (Figure 4): 18 of the *OsPC* genes located on duplicated segments belonged to the same subfamily; the other two *OsPC* genes (*OsUCL1* and *OsSCL2*) present on the duplicated segments between chromosomes 1 and 4 belonged to different subfamilies, with the fourth residue of PCLD involved in copper binding shuffled from Met to Gln. Additionally, 18 genes were found to be tandemly duplicated, and separated by a maximum of five intervening genes. Eight tandemly duplicated genes were present in chromosome 8; four genes were present in each of chromosomes 2 and 3; and two genes in chromosome 4. In all cases of tandem duplications, the *OsPC* genes belonged to the same subfamilies. Interestingly, four groups of *OsUCL* genes (group I: *OsUCL6*, *OsUCL16*, *OsUCL29*, *OsUCL30* and *OsUCL31*; group II: *OsUCL4*, *OsUCL14* and *OsUCL15*; group III: *OsUCL7*, *OsUCL8* and *OsUCL9*; and group IV: *OsUCL10*, *OsUCL11* and *OsUCL21*) and one group of *OsENODL* genes (*OsENODL6*, *OsENODL7* and *OsENODL18*) were expanded through both segmental and tandem duplications.

The duplications of the *PC* gene family in *Arabidopsis* were also investigated (Figure S2). In all cases of duplications, the genes belonged to the same subfamily. The 16 *AtPC* genes (four *AtUC* and 12 *AtENODL* genes) were localized on duplicated segments but only one pair *AtPC* genes (*AtUC3* and *AtUC7*) resulted from tandem duplication. Two groups of *AtENODL* genes (group I: *AtENODL10*, *AtENODL11* and *AtENODL12*; and group II: *AtENODL13*, *AtENODL14* and *AtENODL15*) were expanded through segmental duplications, while one group of *UC* genes (*AtUC2*, *AtUC3* and *AtUC7*) resulted from both segmental and tandem duplications. Compared to duplication events in rice, the segmental duplications contributed most to the expansion of AtPCs.

### Expression profiles of *OsPC* genes during vegetative and reproductive development

We investigated the expression patterns of *OsPC* genes by using three publicly available resources: expressed sequence tag (EST) expression profiles, MPSS tags and microarrays.

The availability of full-length cDNA (FL-cDNA) and ESTs corresponding to *OsPC* genes were surveyed by searching the RAP-DB locus across the UniGene database at NCBI. There was at least one corresponding FL-cDNA and/or EST available for 38 of 62 (61.29%) *OsPC* genes, indicating that a large percentage of these genes were expressed (Table S3). Moreover, the transcriptional abundance of *OsPC* genes was analyzed using the EST data of rice in various organs and tissues, showing tissue-specific expression patterns of several *OsPC* genes: *OsUCL24* in callus; *OsENODL14* and *OsENODL21* in panicles; *OsUCL4* and *OsUCL35* in roots; and *OsUCL15* in stems (Table S3).

**Figure 5:**
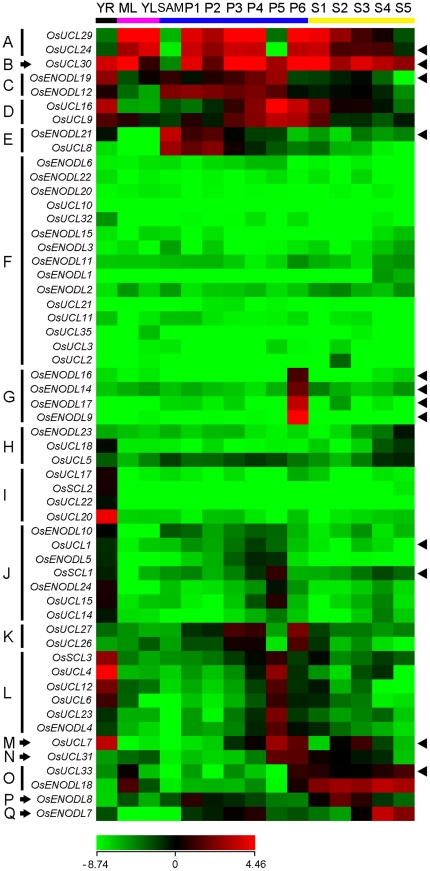
Expression profiles of rice *PC* genes during different vegetative and reproductive developmental stages. The genome-wide microarray data of GSE6893 are reanalyzed. A heat map representing hierarchical clustering of average log signal values of *OsPC* genes in various developmental stages are generated (YR, roots from 7-day-old seedlings; ML, mature leaf; YL, leaves from 7-day-old seedling, different stages of panicle development: SAM, up to 0.5 mm; P1, 0–3 cm; P2, 3–5 cm; P3, 5–10 cm; P4, 10–15 cm; P5, 15–22 cm; P6, 22–30 cm and different stages of seed development: S1, 0–2 dap; S2, 3–4 dap; S3, 5–10 dap; S4, 11–20 dap; S5, 21–29 dap). Genes share similar expression patterns are divided into17 clusters: (A) ML, YL, P1–P4, P6 and S1–S4; (B) all examined organs and tissues except SAM; (C) YR, SAM and P1–P5; (D) YR, P3–P6, and S1; (E) SAM and P1–P3; (F) low expression in all examined organs and tissues; (G) P6; (H) YR, S4 and S5; (I) YR; (J) YR, P4 and P5; (K) P3, P4 and P6; (L) YR and P5; (M) YR, P4–P6, S2 and S3; (N) P5, P6, S1 and S2; (O) ML, P6 and S1–S5; (P) P1 and S1–S3; (Q) P3, P4 and S3–S5. The colour scale (representing average log signal values) is shown at the bottom.

Microarrays provide a high-throughput means to analyze the expression of genes of interest at transcription level. The expression patterns of *OsPC* genes were analyzed using microarray data from an earlier study [17]. Various developmental stages of rice organs and tissues were selected for microarray analysis including young root (YR), mature leaf (ML), young leaf (YL), shoot apical meristem (SAM), and various stages of panicle (P1–P6) and seed (S1–S5) development (Table 2). On Affymetrix rice whole-genome arrays (GPL2025), 56 of 62 *OsPC* genes had at least one probe. A hierarchical cluster display of average log signal-values of these genes was produced (Table S4). The microarray analysis revealed that most *OsPC* genes were expressed in at least one reproductive or vegetative developmental stage (Figure 5). *OsUCL24*, *OsUCL29* and *OsUCL30* were abundantly expressed in all examined organs and tissues except SAM (Figure 5A and B); and *OsENODL12*, *OsENODL19*, *OsUCL16* and *OsUCL9* are highly expressed in YR, panicles and seeds (Figure 5C and D). *OsENODL21* and *OsUCL8* were highly expressed in SAM and early stages of panicle development (P1–P3) (Figure 5E). The expression levels of 15 genes were relatively low in all examined organs and tissues (Figure 5F). Interestingly, four *OsENODL* genes (*OsENODL9*, *OsENOD14*, *OsENOD16* and *OsENOD17*) were specifically expressed in panicles at the stage of pollen maturation (P6) (Figure 5G). *OsENODL23*, *OsUCL18* and *OsUCL17* were mainly expressed in YR and late stages of seed development (S4 and S5) (Figure 5H). It was noteworthy that four genes (*OsUCL17*, *OsSCL2*, *OsUCL22* and *OsUCL20*) were specifically expressed in YR (Figure 5I). Thirteen genes were mainly expressed in YR and panicles at the stage of vacuolated pollen (P5) (Figure 5J and L). *OsUCL26* and *OsUCL27* were mainly expressed in panicles at stages P3, P4 and P6 (Figure 5K); *OsUCL7* was expressed in YR, P4–P6, S2 and S3 (Figure 5M), and *OsUCL31*, *OsUCL33* and *OsENODL18* were mainly expressed in leaves and S1–S5 (Figure 5N and O). *OsENODL7* and *OsENODL8* were mainly expressed in panicles and seeds (Figure 5P and Q).

**Table 2:** Different stages of rice panicle and seed development.

Symbol	Length and DAP^a^	Stage
SAM	up to 0.5 mm^b^	shoot apical meristem
P1	0–3 cm^c^	floral transition and floral organ development
P2	3–5 cm	meiosis
P3	5–10 cm	meiosis
P4	10–15 cm	young microspore
P5	15–22 cm	vacuolated pollen
P6	22–30 cm	mature pollen
S1	0–2 DAP	early globular embryo
S2	3–4 DAP	middle and late globular embryo
S3	5–10 DAP	embryo morphogenesis
S4	11–20 DAP	embryo maturation
S5	21–29 DAP	dormancy and desiccation tolerance

a , DAP, day after pollination.

b , mm, millimetre.

c , cm, centimetre.

MPSS generates thousands of molecules per reaction and provides a sensitive quantitative measure of gene expression for nearly all genes of the genome [18]. Both of 17- base and 20-base signatures from eight different organs and tissues of rice were extracted by searching the RGAP locus across the rice MPSS database, revealing that the MPSS signatures for 31 *OsPC* genes in at least one of the libraries were available (Table S5). This further supports our results that most *OsPC* genes were expressed. Expression differences were displayed by estimating the number of tags of *OsPC* genes (tpm, transcripts per million): low was <50 tpm, moderate was 50–500 tpm, and strong was >500 tpm. There were eight *OsPC* genes expressed at a high level, and 15 and eight at moderate and low levels, respectively (Table S5). The differential expression of *OsPC* genes in different MPSS libraries was investigated. Six *OsPC* genes were identified with tissue-specific expression patterns: *OsENODL8* in ovary and mature stigma, *OsENODL9* and *OsENODL14* in mature pollen, *OsUCL20* and *OsUCL35* in 14 day-old young roots, and *OsUCL27* in 14 day-old young leaves. Another six genes showed highly abundant expression levels: *OsENODL2* in mature pollen, *OsENODL5* and *OsENODL19* in immature panicle, *OsUCL1* in 60 day-old stem, *OsUCL4* in 14 day-old young roots, and *OsUCL8* in ovary and mature stigma. (Table S5).

**Figure 6:**
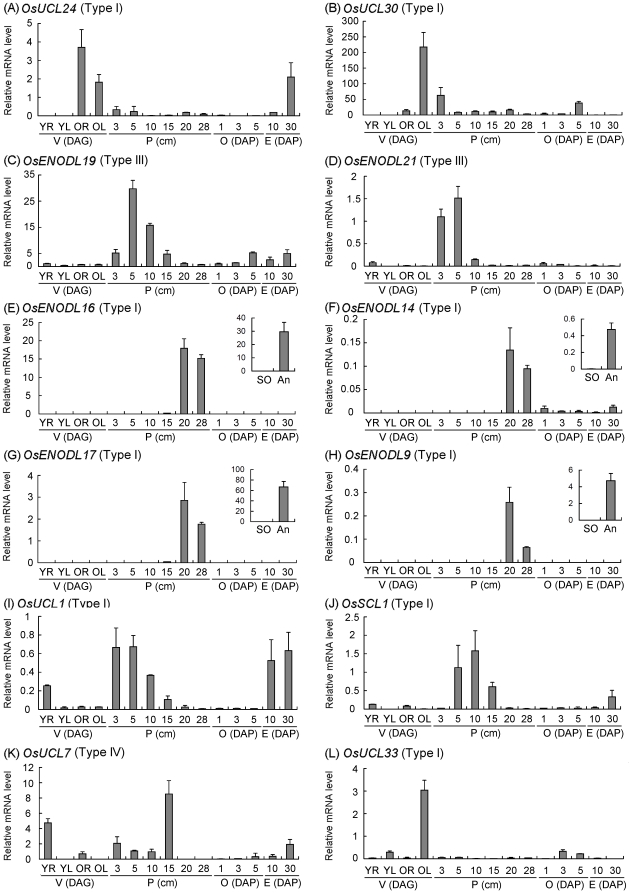
Real-time PCR verification of the expression of representative rice *PC* genes in different developmental stages of vegetative and reproductive tissues and organs. YR and YL, 7-day-old roots and leaves; P1–P6, <3, 5, 10, 15, 20, and 28 cm panicles; O1–O3, 1, 3 and 5 DAP ovaries; E1 and E2, 10 and 30 DAP embryos; An and SO, anthers and stigmas and ovaries from 28 cm panicles. Error bars indicate standard deviations of independent biological replicates (n = 2 or more).

To validate the results of digital expression analysis, qRT-PCR was performed for several representative genes. The expression patterns of selected genes in roots, leaves and panicles were in general agreement with the data of microarrays and MPSS tags. For example, *OsENODL19* and *OsENODL21* were expressed during panicle development (Figure 6C and D); *OsENODL16*, *OsENODL14*, *OsENODL17* and *OsENODL9* were specifically expressed in 20 and 28 cm panicles (Figure 6E–H); *OsSCL1* and *OsUCL7* were predominantly expressed in roots and 15 cm panicles; and *OsUCL33* was highly expressed in mature leaves [60 days after germination (DAG)]. In our qRT-PCR analysis, different stages of seed development were replaced by ovaries of 1, 3 and 5 days after pollination (DAP) seeds and embryos of 10 and 30 DAP seeds. This may have caused the difference in the results of microarray analysis in seeds and qRT-PCR analysis in ovaries and embryos. For instance, *OsUCL24* was mainly expressed in mature roots and leaves and *OsUCL30* in mature leaves, 3 cm panicle and 5 DAP ovaries, which differed from the microarray results (Figure 6A and B). Moreover, to better dissect the expression of four *ENODL* genes that were specifically expressed in mature panicles (*OsENODL9*, *OsENODL14*, *OsENODL16* and *OsENODL17*), we investigated their expression in stigma, ovary and anther from 28 cm panicles (Figure 6). The four genes were preferentially expressed in anthers compared to stigma and ovary.

### Expression profiles of *OsPC* genes in response to abiotic stresses

**Figure 7:**
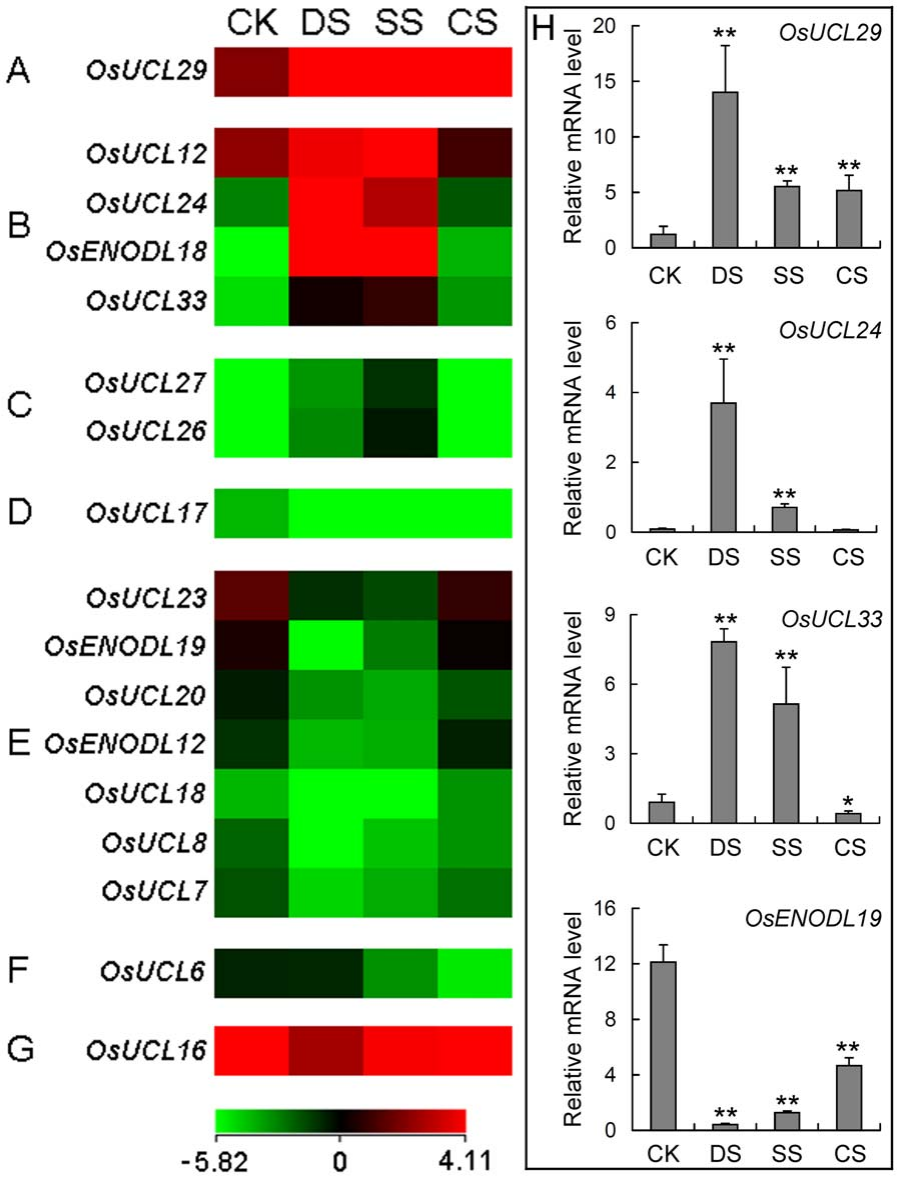
Expression profiles of rice *PC* genes differentially expressed under the treatments of abiotic stresses. The genome-wide microarray data of GSE6901 are reanalyzed. A heat map representing hierarchical clustering of average log signal values of *OsPC* genes under control and various stress conditions are generated (CK, control; DS, drought stress; SS, salt stress; CS, cold stress). Genes that exhibited two-fold or more differential expression are shown. (A) Up-regulated by drought, salt and cold stresses; (B) Up-regulated by drought and salt stresses; (C) Up-regulated by salt stress; (D) Down-regulated by drought, salt and cold stresses; (E) Down-regulated by drought and salt stresses. (F) Down-regulated by salt and cold stresses; (G) Down-regulated by drought stress; (H) Differentially expressed *OsPC* genes are selected for qRT-PCR analyses. The significance of difference between the controls and treatments are determined by Origin 7.5, two asterisks represent (**, P<0.01) and one asterisk represents (*, 0.01<P<0.05). The colour scale representing average log signal values is shown at the bottom. Error bars indicate standard deviations of independent biological replicates (n = 4 or more).

When plants were subjected to environmental stresses, various physiological and biochemical responses were induced, leading to changes in gene expression. To investigate the response of *OsPC* genes to abiotic stresses, the microarray results from 7-day-old seedlings subjected to drought, salt and cold stresses were analyzed (Figure 7). A total of 17 genes were significantly (*P*<0.05) down- or up-regulated (<0.5 or >2) compared to controls in at least one of the stress conditions examined (Table S4). The transcriptional levels of *OsUCL29* and *OsUCL17* were up- and down-regulated by all three stresses, respectively (Figure 7A and D); four genes (*OsUCL12*, *OsUCL24*, *OsENODL18* and *OsUCL33*) were up-regulated and seven genes (*OsUCL23*, *OsENODL19*, *OsUCL20*, *OsENODL12*, *OsUCL18*, *OsUCL8* and *OsUCL7*) were down-regulated by drought and salt stresses (Figure 7B and E); two genes (*OsUCL27* and *OsUCL26*) were up-regulated by salt stress (Figure 7C); *OsUCL6* was down-regulated by salt and cold stresses (Figure 7F); and *OsUCL16* was down-regulated by drought stress (Figure 7G).

Moreover, the expression levels of four representative genes were investigated using qRT-PCR under various stress conditions imposed for 3 h (Figure 7H). The qRT-PCR results agreed well with the microarrays. The expressions of *OsUCL29* were induced by all three abiotic stresses, *OsUCL24* was up-regulated by drought and salt stresses, *OsUCL33* was up-regulated by drought and salt stresses but down-regulated by cold stress, and *OsENODL19* was significantly down-regulated by drought and salt stresses. This suggested that *OsPC* genes might play a significant role in abiotic stress pathways and be a valuable resource for investigating stress tolerance in rice.

### Expression comparison between *OsPC* and *AtPC* genes

**Figure 8:**
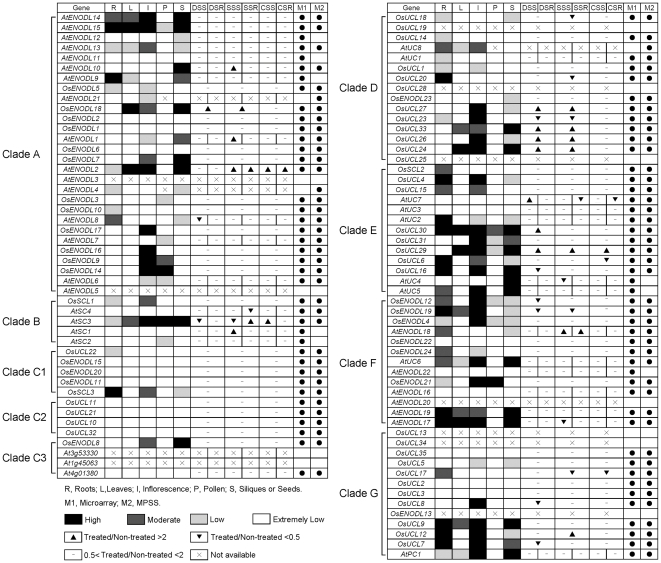
Expression comparison between rice and *Arabidopsis PC* genes in different developmental stages of tissues and organs and under the treatments of abiotic stresses. The *OsPC* and *AtPC* genes are arranged according to the same order of phylogenetic tree (Figure 3). The expression data of microarrays and MPSS tags are used for expression analysis. M1 and M2 represent microarrays and MPSS tags, respectively. White, light grey, dark gray, and black boxes indicate extremely low (less than 0.5 or no signature is found), low (between 0.5 and 1 or the signature numbers between 0 and 50 tpm), moderate (between 1 and 2 or between 50 and 500 tpm), and high (more than 2 or more than 500 tpm) expression levels, respectively. The symbol “×” represents no probe or signature on microarray and MPSS. R, root; L, leaf; I, inflorescence; S, silique or seed; DSS and DSR; drought stressed shoot and root; SSS and SSR, salt stressed shoot and root; CSS and CSR, cold stressed shoot and root.

To investigate the expression of both *OsPC* and *AtPC* genes, a comparative expression analysis was conducted using the microarray and MPSS data in root, leaf, inflorescence, pollen, silique/seed and under abiotic stresses (Figure 8 and Tables S4 and S5). Six *OsPC* and five *AtPC* genes were absent from the two data sets. Integrating the data of microarray and MPSS tags, it was showed that 49 *PC* genes were expressed in more than two organs and tissues examined and 18 *PC* genes acted in tissue-specific manners (Figure 8). The expression levels of 22 *PC* genes were extremely low in all examined organs and tissues (Figure 8). This analysis revealed that 24 *PC* genes with close evolutionary relationships had similar expression patterns. For instance, *AtENODL10*, *OsENODL18*, *AtENODL1* and *AtENODL2* were significantly up-regulated by abiotic stresses and mainly expressed in inflorescences and seeds. Moreover, it was noteworthy that six genes (*OsENODL17*, *AtENODL7*, *OsENODL16*, *OsENODL9*, *OsENODL14* and *AtENODL6*) were specifically expressed in inflorescences and/or pollen. In addition, the expressions of 32 *OsPC* genes differed from those of their *Arabidopsis* homologs. For example, the expression of the SC subfamily differed between rice and *Arabidopsis*; *AtSC3* were highly expressed but *AtSC1*, *AtSC2* (except in pollen) and *AtSC4* had extremely low expression in all examined organs and tissues. However, *OsSCL1* was mainly expressed in roots and inflorescences; *OsSCL2* in roots; and *OsSCL3* in roots, inflorescences and seeds.

Interestingly, 15 of 20 gene pairs located on duplicated chromosomal segments and 9 of 13 tandem duplicated gene pairs showed differential expression patterns, suggesting that most duplicated gene pairs were under the diverse transcriptional controls (Figures 8 and S3). This finding was similar to previous results showing that the expression of duplicated genes frequently diverges compared to that of their ancestors, suggesting that duplication was a major reason for the enrichment of the functions of this family during the long course of evolution [19]–[21].

## Discussion

### PC-like AGPs in rice and *Arabidopsis*

In recent years, several bioinformatics approaches were utilized to identify the *AGP* gene family in rice and *Arabidopsis*, e.g. calculating the PAST amino acid bias for AGPs in rice and *Arabidopsis* [13], [22], using a well-designed BIO OHIO program for HRGPs in *Arabidopsis* [12], and BLAST searches for ENODLs in *Arabidopsis* [14]. By these approaches, a large number of classical AGPs, lys-rich AGPs, AG-peptides, non-specific lipid transfer protein-like AGPs (nsLTP-like AGPs) and fasciclin-like AGPs were identified in rice and *Arabidopsis* [13], [14]. Similar to the nsLTP-like and fasciclin-like AGPs, typical PC-like AGPs have a SP, AG glycomodules and a GAS in addition to PCLDs. Previously, three ENOD-like AGPs were identified based on biased amino acid composition, namely OsENODL1 (OsELA1), OsENODL6 (OsELA2) and OsENODL18 (OsELA3) [13]. In the present study, we identified 19 UC-like, 1 SC-like and 18 ENOD-like AGPs in rice. It has also been reported that there were 18 ENOD-like AGPs in *Arabidopsis* [14]. However, up to now, the members of UC-like AGPs and SC-like AGPs had not been investigated. We found seven UC-like and four SC-like AGPs in *Arabidopsis*. The number of ENOD-like AGPs was the same among the two species and there were many more UC-like AGPs in rice compared to *Arabidopsis* (19 and 7, respectively). Although a large number of PC-like AGPs had been identified in rice and *Arabidopsis* with bioinformatics advances, only two ENODLs were found to be reactive with β-GlcY in rice and *Arabidopsis* [7], [8]. Thus, more biochemical evidences of glycosylation and GPI-anchor modification were required to identify PC-like AGPs at the protein level.

### Duplications play major roles in diversification of the *OsPC* gene family

Gene duplications represent the major mechanism for gene family expansion through either chromosomal segment duplications or tandem duplications during evolution [23]. In the present study, the gene duplication analysis revealed that 38 of 62 *OsPC* genes resulted from duplications, of which, 33 duplicated *OsPC* genes coded for OsUCLs, indicating that the duplication events within the OsUCL subfamily contributed to most of the expansion of the *OsPC* gene family. Moreover, segmental (20 genes) and tandem (18 genes) duplications contributed almost equally to the expansion of the *OsPC* gene family.

A change in a duplicated locus might not exhibit morphological and/or physiological phenotypes, but might contribute to diverse developmental events. When gene duplication occurs, some genes may retain their original functions and expression patterns. For example, *OsENODL12* and *OsENODL19* localized on duplicated segments exhibit similar expression patterns in both development stages and abiotic stresses, indicating their overlapping functions (Figures 5C and S3C). And in other cases, some genes may be located behind a new series of regulatory elements through acquisition and/or deletion of regulatory sequences, such as tissue-specific enhancers and stress induced elements: e.g. *OsUCL23*, *OsUCL24*, *OsUCL26* and *OsUCL27* were tandemly duplicated genes. The expression of *OsUCL26* and *OsUCL27* had similar expression patterns in panicles (P3, P4 and P6) and were up-regulated under salt stresses (Figures 5K, 7C and S3E), however, *OsUCL24* expression was high in all organs and tissues, except SAM, P5 and S5, and was up-regulated under drought and salt stresses (Figures 5A, 7B and S3E). Additionally, the expression of *OsUCL23* exhibited divergent expression patterns, it was highly expressed in P5 and down-regulated under drought and salt stresses (Figure 5L, 7E and Figure S3E). The expression analysis of duplicated *OsPC* genes suggested that duplication events in this gene family might have led to sub- or neo-functionalization, which might have contributed the most to the functional diversification of the *OsPC* gene family.

### Biased expression patterns of *OsPC* genes derived from ESTs, microarrays and MPSS

In the present study, multiple high-throughput gene expression datasets from ESTs, microarrays and MPSS platforms were integrated to generate a transcriptome atlas of the *OsPC* gene family. The expression data of most *OsPC* genes was in general agreement between different datasets. However, the EST data of several genes were not consistent with microarray or MPSS analyses. For instance, *OsUCL24* was expressed in callus, leaves and stem according to EST data; in leaves, panicles and seeds according to microarray data; and in roots and callus according to MPSS data. There were also other examples that the expression of one gene from these datasets was not consistent (Tables S3, S4, S5), and the reasons for this might be complicated. Although various tissues and organs were collected for these high-throughput expression analyses, their developmental stages were not always consistent. For example, 7- and 14-day-old roots and leaves were selected for analysis in microarray and MPSS, respectively.

Although these high-throughput transcriptome advances provided genomic overviews of gene expression, there were still some disadvantages. The EST profiles show approximate gene expression patterns as inferred from EST counts and the cDNA library sources, which presents limitations for a variety of reasons, such as different methods being used for normalization and subtraction. Microarrays and MPSS provide more accurate expression estimation than EST profiles, but they also have limitations that cannot be overcome. The sensitivity of microarray analysis is still low despite the improvements in signal detection [24]; it can only detect genes with high expression levels and is unsuited to investigate genes with low expression levels. In the present study, the expressions of 15 *OsPC* genes were relatively low compared to others in microarrays, but some were detectable in MPSS (Figure 5F and Table S5). The number of DNA probes on a microarray plate was also limited, six *OsPC* genes were absent from the popular microarray plate for rice (GPL2025). Although MPSS overcomes the detection of weakly expressed transcripts and limitation of DNA probes, it cannot distinguish between closely related sequences. Additionally, due to the nucleotides bias, some transcripts were lost in the course of library construction [25]. Therefore, qRT-PCR confirmation of the expression of *OsPC* genes is necessary. The expression of *OsUCL29* was similarly induced by drought, salt and cold stresses in microarrays (Figure 7A). However, the expression level of *OsUCL29* was greater under drought than under salt and cold stresses in qRT-PCR. Compared to these high-throughout means, the qRT-PCR technique is more sensitive and accurate for investigating gene expression.

### Roles of PCs in pollen development and in response to abiotic stresses

Our transcriptional analyses revealed that four *OsENODL* genes (*OsENODL9*, *OsENODL14*, *OsENODL16* and *OsENODL17*) were specifically expressed in panicles at the stage of mature pollen (20–30 cm panicles), especially in anther and/or pollen compared to stigma and ovary (Figures 6 and 8, and Tables S3 and S5). This observation was consistent with their homologues in *Arabidopsis*, *AtENODL5* and *AtENODL6*, which were also differentially expressed in mature pollen (Figure 8) [26]. The functions of pollen-specific AGPs have been discussed in the previous reports [26]–[28]. Down-regulation of two classical AGPs (*AtAGP6* and *AtAGP11*) specifically in pollens and pollen tubes leads to reduced fertility, indicating that *AtAGP6* and *AtAGP11* play an important role in early germination and pollen tube growth [27]. AtFLA3 with similar structure to ENODLs is related to microspore development and affected pollen intine formation [28]. Due to the presence of AG modifications and the GPI-anchor attachment, it was proposed that PCs function in cell-to-cell signaling, cell differentiation, and signal transduction pathways [29]. *OsENODL9*, *OsENODL14*, *OsENODL16* and *OsENODL17* encode type I OsPCs and are regarded as chimeric AGPs. The loss or substitution of the key amino acid for copper binding is a common feature of the ENODL subfamily, underlying their possible functions other than copper binding. However, the detailed expression patterns and functions of the four *OsENODL* genes during anther and/or pollen development require further exploration.

Interestingly, six *OsUCL* genes were up-regulated by drought and salt stresses (Figure 7A–C). When plants were subjected under drought and salt stresses, over-reduction of the electron transport chain was induced and had the potential to produce oxidative stress [30]. Hydrogen peroxide and the superoxide radical are mainly formed by the electron transport systems of chloroplasts and mitochondria [31]. The *PC* gene family had been related to the transfer of electrons in photosynthesis [2]. It was reported that an *Arabidopsis* blue copper binding protein (AtBCB/AtSC3) was induced by aluminum stress and oxidative stress [32], [33]. Over-expression of the *AtBCB/AtSC3* gene in *Arabidopsis* conferred aluminum resistance [34], [35]. The transformed onion cells carrying the *AtBCB::GFP* showed a stronger signal in the plasma membrane (PM) but a lower signal in the cytoplasm [5]. Though most PCs were shown to be PM-localized proteins, the involvement of PCs in electron transfer of photosynthesis suggests that there may be some relevancy between the functions of PCs and abiotic stresses.

### Conclusions

In our study, 62 members of the *OsPC* gene family were identified and extensive expression analyses of the OsPCs were performed. The *OsPC* and *AtPC* genes were sorted into three subfamilies belonging to six types, supported by phylogeny and protein motifs. The fact that the majority of subfamilies contained members from both rice and *Arabidopsis* suggested that the functions of most of *PC* genes were conserved during angiosperm evolution. In addition, gene duplication analysis revealed that the OsUCL subfamily had expanded more than that of *Arabidopsis*, showing both the conservation and divergence of gene function. Extensive expression analysis indicated that *OsPC* genes were preferentially expressed in roots, panicles and seeds, some of them especially in anther and/or pollen. In the future, reverse genetic methods, such as RNA interference and mutant identification, should be utilized to investigate the functions of interesting genes. The present study has established a solid foundation for functional research of the *OsPC* gene family and has improved our understanding of the functions of PCs in monocots.

## Materials and Methods

### Plant materials and treatment methods

*Oryza sativa* L. *japonica cv. Nipponbare* were grown in a greenhouse at Wuhan University, the temperature for plant growth was 30/28°C under a photoperiod of 16 h light and 8 h dark. Young roots and leaves were collected from 7-day-old seedlings growing in containers with sponges as supporting materials in sterile water. Plant materials spanning all stages of vegetative (root and leaf) and reproductive development (panicle and seed) were collected from flowering rice plants. For stress treatments, 7-day-old seedlings were carefully transferred onto filter papers as drought stress, placed in 400 mM NaCl solution as salt stress, and kept at 4°C as low temperature stress, respectively. 7-day-old seedlings placed in deionized water as control. The treatments of control, drought and salt stresses were placed in a 28°C illumination incubator. Three seedlings with similar height were selected for each of five independent biological replicates. The duration of all stress treatments is 3 h and the fresh material of each sample before and after drought treatments were weighed to determine the degrees of water loss. The average degree of water loss was 43.95%. Besides, two additional biological replicates under drought stress were placed into deionized water for 1 day recovery. In our observation, the seedlings after drought treatments were moderately stressed and allowed to recover. All materials described above were taken and quickly frozen in liquid nitrogen and stored at −80°C until RNA extraction.

### Identification of OsPCs and bioinformatics analysis

Name searches and BLAST (Basic Local Alignment Search Tool) searches across the rice protein databases were employed to identify the *OsPC* genes from rice genome. Name searches were performed using “plastocyanin”, “uclacyanin”, “stellacyanin” and “early nodulin” as keywords in RGAP (Rice Genome Annotation Project, http://rice.plantbiology.msu.edu/) and RAP-DB (The Rice Annotation Project Database, http://rapdb.dna.affrc.go.jp/). The Hidden Markov Model (HMM) profile of plastocyanin-like domain were downloaded from Pfam (http://pfam.sanger.ac.uk/) (PF02298) in fasta format and were used to screen the protein databases in RGAP and RAP-DB using BLASTP with default settings. After removing the redundant sequences, the remaining candidates were subjected to InterProScan (http://www.ebi.ac.uk/Tools/InterProScan/) to confirm the existence of plastocyanin-like domain (PCLD).

All OsPCs were subjected to SignalP 3.0 (http://www.cbs.dtu.dk/services/SignalP/), Big-PI Plant Predictor (http://mendel.imp.ac.at/gpi/plant_server.html), and NetNGlyc 1.0 Server (http://www.cbs.dtu.dk/services/NetNGlyc/) to check the presence of N-terminal signal peptide, GPI-anchor modification signal, and N-glycosylation sites, respectively. Putative AG glycomodules were predicted mainly following the criterion described before [22], Pro residues are considered to be hydroxylated and arabinoglactosylated if they contained predominantly (Ala/Gly/Ser/Thr)-Pro throughout the Pro-rich region with no more than 11 amino acid residues between consecutive Pro residues. Besides, (Ala/Ser/Thr)-Pro_2–4_ arranged in a noncontiguous manner were defined as putative arabinosylation site [36]–[39]. The structure characteristics of OsPCs and AtPCs were shown in Table S2.

### Sequence and phylogenetic analysis

Plastocyanin-like domains (PCLDs) from all the amino acid sequences of OsPCs and AtPCs were aligned using Clustal X (version 1.83) program. An un-rooted phylogenetic tree was constructed in Clustal X using neighbor-joining method with default parameters. Bootstrap analysis was performed using 1000 replicates, and the tree was visualized using Treeview program.

A Bayesian estimation of phylogeny between OsPCs and AtPCs was also performed using the MrBayes (http://mrbayes.csit.fsu.edu/index.php). The simulation technique MCMC (Markov chain Monte Carlo) was used to approximate the posterior probabilities of trees. The file of PCs.nex was generated by MEGA 4.0 using an input file in fasta format which included the amino acid sequences of OsPCs and AtPCs. Consecutive commands such as “execute PCs.nex”, ““prset aamodelpr = mixed” and “mcmc ngen = 1000000 samplefreq = 1000” were executed. Thirty million times calculation was performed until “the average standard deviation of split frequencies” was less than 0.01. The program was stopped by typing “sump burnin = 25” and the phylogenetic tree was visualized using Treeview program.

### Chromosomal localization and gene duplications

The chromosomal locations of *OsPC* genes were mapped on the physical maps of rice chromosomes by BLASTN search. Genes separated by five or fewer genes were considered to be tandem duplicates. Genes belonging to segmental duplicates were detected by searching the “Segmental genome duplication of rice” in RGAP database (http://rice.plantbiology.msu.edu/segmental_dup/index.shtml). For *Arabidopsis*, the locations of *AtPC* genes were determined on the five *Arabidopsis* chromosomes using the Chromosome Map Tool at TAIR website. The presences of *AtPC* genes on duplicated chromosomal segments were investigated using “Paralogous in *Arabidopsis*” with the default parameters set (http://wolfe.gen.tcd.ie/athal/dup) (Figure S2).

### Digital expression analysis

EST (expressed sequence tag) expression profiles of *OsPC* genes were obtained from UniGene database at NCBI. Genes were defined as specifically expressed if the EST number of any tissue contributed more than half of the total frequency (Table S3).

The results of rice microarrays were available at the Rice Functional Genomic Express Database (http://signal.salk.edu/cgi-bin/RiceGE). Different stages of panicle and seed development were categorized for temporal and spatial expression analysis according to panicle length and days after pollination (Table 2) [17], [40]. Rice seedlings were transferred to a beaker containing 200 mM NaCl solution for salt stress, dried between folds of tissue paper at 28±1°C in a culture room for drought stress, and kept at 4±1°C for cold stress, for 3 h treatment respectively [17]. The expression data of *Arabidopsis* from different developmental stages comparable to those used for rice were downloaded from “Bulk Gene Download” at Nottingham Arabidopsis Stock Centre (http://affymetrix.arabidopsis.info/narrays/help/psp-wbubn.html). The results of developmental stages (GSE5629–5633) and abiotic stresses treatments (GSE5620–5621 and 5623–5624) were used to analyze the expression patterns of *AtPC* genes. For all microarray analysis in rice and *Arabidopsis*, if more than one probe set was available for one gene, the probe set designed from 3′ end was given preference. In order to make these absolute signal values suitable for cluster display, the absolute values were divided by the average of all absolute values. Hierarchical cluster displays were generated from the logarithmic values of the ratios in previous step, using Cluster and Treeview (Table S4) [41].

MPSS tags of *OsPC* and *AtPC* genes were obtained from the MPSS project (http://mpss.udel.edu) mapped to TIGR and TAIR gene models, respectively. The signature was considered to be significant if it uniquely identified an individual gene and showed perfect match (100% identity over the tag length). The normalized abundance (tpm, tags per million) of these signatures for a given gene in a given library represented a quantitative estimate for the expression of that gene. MPSS expression data for 17- and 20-base signatures representing eight different organs and tissues were used for the analysis (Table S5).

### Quantitative real-time RT-PCR analysis

Quantitative real-time RT-PCR (qRT-PCR) was carried out by SYBR-green fluorescence using a Rotor-Gene Q real-time PCR machine (Qiagen). Gene-specific primers were designed for all the OsPC genes preferentially from 3′ end of the gene using Primer 3 with default parameters (http://frodo.wi.mit.edu/primer3/) (Table S6). At least two and four independent biological replicates and three technical replicates of each biological replicate were made for qRT-PCR analysis in developmental stages and abiotic stresses, respectively. Total RNA was isolated from each sample with Plant RNA Purification Reagent (Invitrogen). The qRT-PCR were performed as described previously with some modifications [42].The RNA samples were incubated at 37°C for 30 min with DNase I (RNase-free) (Fermentas, #EN0521) to remove the DNA contamination prior to RNA reverse transcription. Then, 1 µl EDTA (50 mM) was added to the mixture and incubated at 65°C for 10 min to inactive DNase I. The first-strand cDNA was synthesized from 1 µg total RNA using reverse transcriptase (ReverTra Ace, TOYOBO); 10-fold diluted cDNA samples were used for qRT-PCR. The primer specificity was further confirmed by dissociation curve analysis obtained after the real-time PCR. The expression of each gene in different RNA samples was normalized using the geometric average of the expression levels of four frequently-used housekeeping genes: *UBQ5*, *eEF-1α*, *18S rRNA* and *25S rRNA* [43]–[45]. The relative expression levels were analyzed using the standard curve method, three times diluted series of a mixed cDNA pools were selected to build a stand curve for each gene. The given values of these diluted series are 10, 30, 90, 270, 810 and 2430 (from low to high).

## Supporting Information

Figure S1 **Bayesian phylogenetic analysis of rice and** ***Arabidopsis*** **PCs using Mr Bayes program.** Values at the internodes are posterior probability for MrBayes reconstructions.
(TIF)

Figure S2 **Chromosomal localization of** ***Arabidopsis PC*** **genes.** Chromosome numbers are indicated at the top of each chromosome. Genes presented on duplicated segments of genome are connected by blue lines, and tandem duplicated genes are marked by a vertical bar.
(TIF)

Figure S3 **Expression analysis of duplicated rice** ***PC*** **genes.** Expression patterns are analyzed for duplicated *OsPC* genes found in segmental and tandem duplication of rice genome. X-axis represents the developmental stages. Y-axis represents the raw expression values obtained from microarray.
(TIF)

Table S1 **List and characteristics of rice PCs.**
(DOC)

Table S2 **Protein backbones of rice and Arabidopsis PCs.**
(DOC)

Table S3 **ESTs expression profiles of rice** ***PC*** **genes.**
(XLS)

Table S4 **Microarray analysis of rice and** ***Arabidopsis PC*** **genes.**
(XLS)

Table S5 **MPSS analysis of rice and** ***Arabidopsis PC*** **genes.**
(XLS)

Table S6 **Primers used in qRT-PCR of rice** ***PC*** **genes.**
(DOC)
