# Supplementary figures and images for: The Phytocyanin Gene Family in Rice (Oryza sativa L.): Genome-Wide Identification, Classification and Transcriptional Analysis
Source: PLoS One. 2011 Oct 3;6(10):e25184. doi: 10.1371/journal.pone.0025184 (PMC3184959; doi:10.1371/journal.pone.0025184)

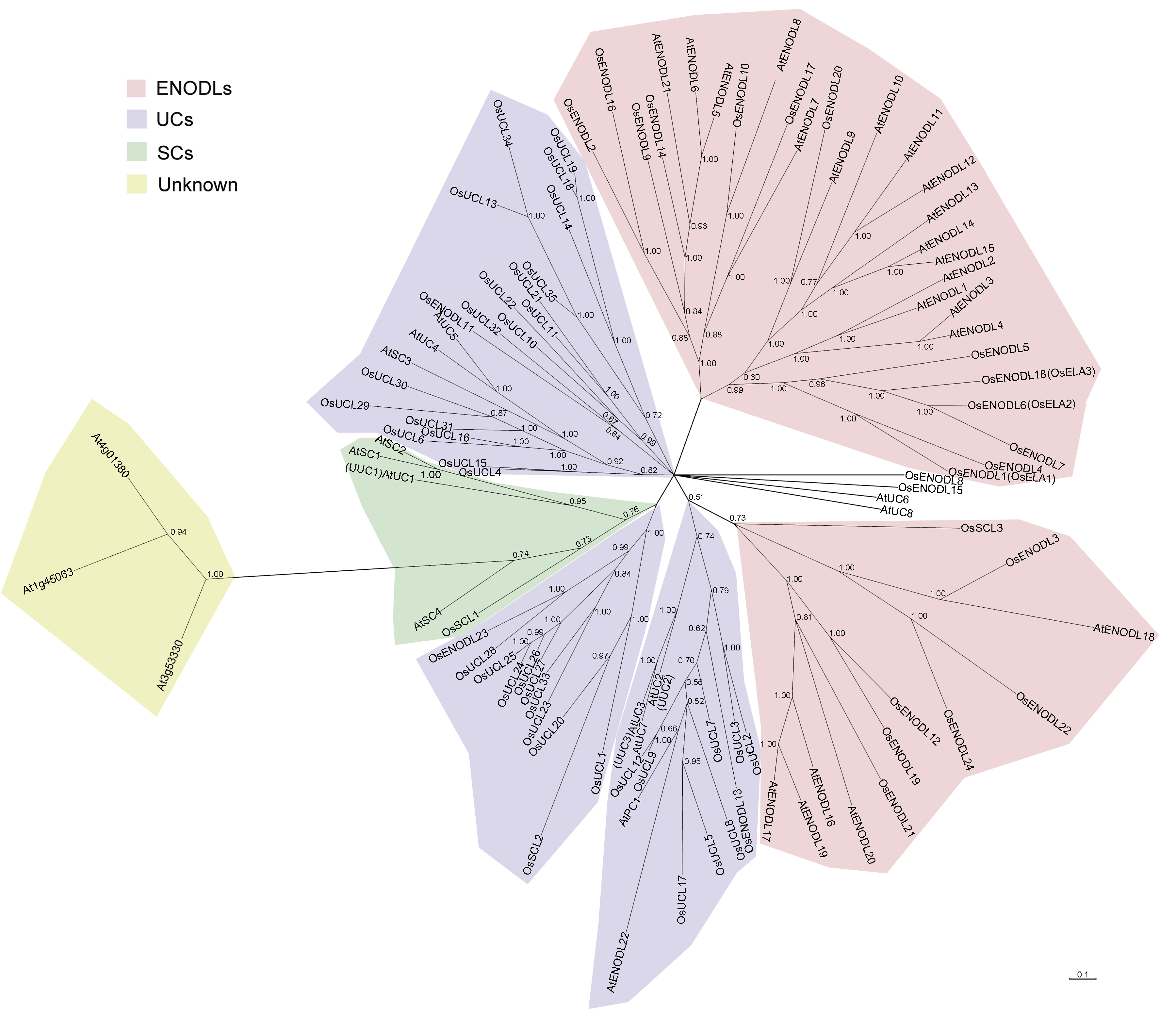

Supplement: Figure S1 — Bayesian phylogenetic analysis of rice and Arabidopsis PCs using Mr Bayes program. Values at the internodes are posterior probability for MrBayes reconstructions. (TIF) [file pone.0025184.s001.tif]

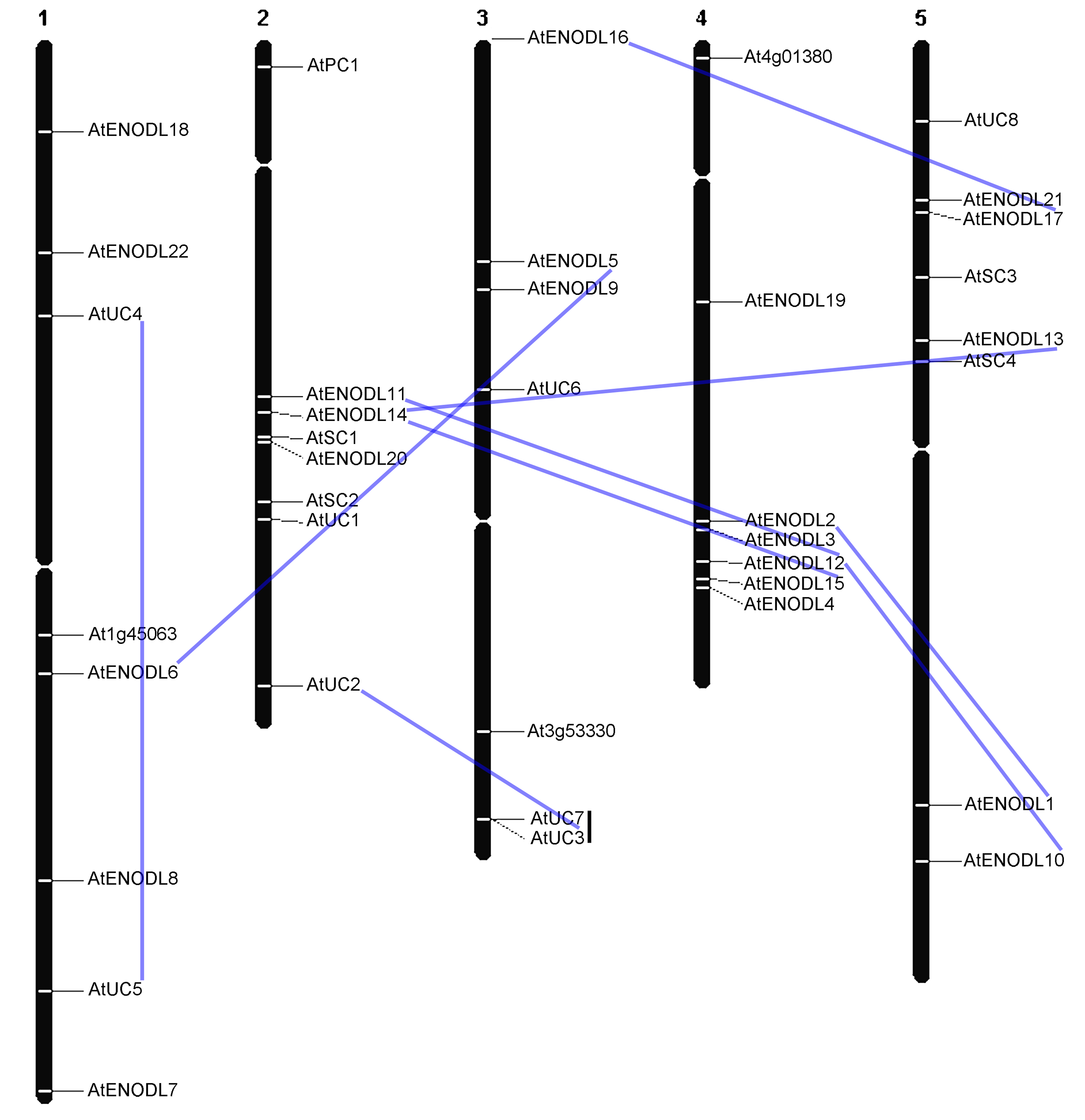

Supplement: Figure S2 — Chromosomal localization of Arabidopsis PC genes. Chromosome numbers are indicated at the top of each chromosome. Genes presented on duplicated segments of genome are connected by blue lines, and tandem duplicated genes are marked by a vertical bar. (TIF) [file pone.0025184.s002.tif]

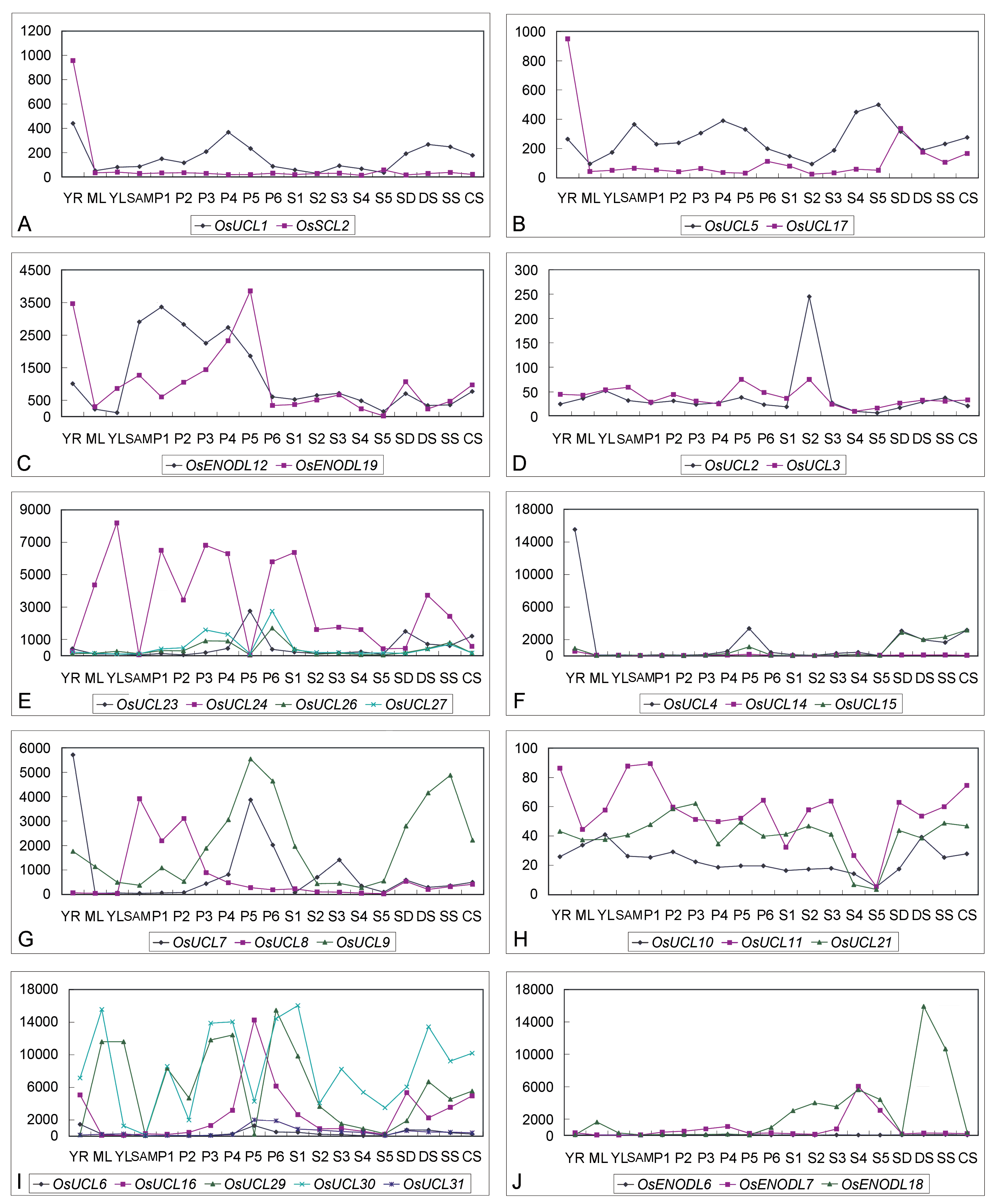

Supplement: Figure S3 — Expression analysis of duplicated rice PC genes. Expression patterns are analyzed for duplicated OsPC genes found in segmental and tandem duplication of rice genome. X-axis represents the developmental stages. Y-axis represents the raw expression values obtained from microarray. (TIF) [file pone.0025184.s003.tif]
