# Supplementary material for: The Phytocyanin Gene Family in Rice (Oryza sativa L.): Genome-Wide Identification, Classification and Transcriptional Analysis
Source: PLoS One. 2011 Oct 3;6(10):e25184. doi: 10.1371/journal.pone.0025184 (PMC3184959; doi:10.1371/journal.pone.0025184)
Supplement: Table S1 — List and characteristics of rice PCs. (DOC) [file pone.0025184.s004.doc]

## Table 1. List and characteristics of rice PCs

| Name^a^ | Type^b^ | RAP-DB^c^ | RGAP^d^ | cDNA^e^ | SP^f^ | GPI^g^ | AG^h^ | N-glyco^i^ | Cu sites^j^ |
| --- | --- | --- | --- | --- | --- | --- | --- | --- | --- |
| **OsENODL1** | I | Os01g0748150 | LOC_Os01g54430 | AK242185 | + | + | + | + | - |
| OsENODL2 | I | Os01g0201500 | LOC_Os01g10480 | NA | + | + | + | + | - |
| OsENODL3 | I | Os01g0272700 | LOC_Os01g16610 | NA | + | + | + | + | - |
| OsENODL4 | I | Os01g0281600 | LOC_Os01g17470 | AK062646 | + | + | + | + | - |
| OsENODL5 | I | Os01g0788700 | LOC_Os01g57880 | C74750 | + | + | + | + | - |
| **OsENODL6** | I | Os02g0162200 | LOC_Os02g06670 | NA | + | + | + | + | - |
| OsENODL7 | I | Os02g0162400 | LOC_Os02g06690 | NA | + | + | + | - | - |
| OsENODL8 | I | Os02g0720100 | LOC_Os02g48820 | AK063724 | + | + | + | - | - |
| OsENODL9 | I | Os02g0725500 | LOC_Os02g49350 | CT835340 | + | + | + | + | - |
| OsENODL10 | I | Os03g0115000 | LOC_Os03g02400 | AK107855 | + | + | + | + | - |
| OsENODL11 | IV | Os03g0648500 | LOC_Os03g44630 | AK107766 | + | - | - | + | - |
| OsENODL12 | III | Os03g0758500 | LOC_Os03g55120 | AK102002 | + | + | + | + | - |
| OsENODL13 | IV | Os04g0422200 | LOC_Os04g34480 | NA | + | - | - | - | - |
| OsENODL14 | I | Os04g0673800 | LOC_Os04g57750 | AK071236 | + | + | + | - | - |
| OsENODL15 | III | Os05g0570900 | LOC_Os05g49580 | C73014 | + | + | - | + | - |
| OsENODL16 | I | Os06g0286228 | LOC_Os06g17730 | AK069113 | + | + | + | + | - |
| OsENODL17 | I | Os06g0553800 | LOC_Os06g36010 | AK072932 | + | + | + | + | - |
| **OsENODL18** | I | Os06g0681200 | LOC_Os06g46740 | AK107980 | + | + | + | + | - |
| OsENODL19 | III | Os07g0112700 | LOC_Os07g02200 | AK073121 | + | + | - | + | - |
| OsENODL20 | I | Os08g0273300 | LOC_Os08g17160 | AK109468 | + | + | + | - | - |
| OsENODL21 | III | Os09g0557900 | LOC_Os09g38540 | AK108807 | + | + | - | + | - |
| OsENODL22 | I | Os11g0491500 | LOC_Os11g29910 | NA | + | + | + | + | - |
| OsENODL23 | I | Os12g0150500 | LOC_Os12g05470 | NA | + | + | + | + | - |
| OsENODL24 | I | Os12g0454600 | LOC_Os12g26880 | CT835529 | + | + | + | + | - |
| OsUCL1 | I | Os01g0786500 | LOC_Os01g57690 | CI165732 | + | + | + | - | + |
| OsUCL2 | IV | Os02g0256800 | LOC_Os02g15710 | NA | + | - | - | - | + |
| OsUCL3 | IV | Os02g0257100 | LOC_Os02g15730 | AK242540 | + | - | - | - | + |
| OsUCL4 | I | Os02g0653200 | LOC_Os02g43660 | AK062889 | + | + | + | - | + |
| OsUCL5 | IV | Os02g0731400 | LOC_Os02g49850 | AK243067 | + | - | - | - | + |
| OsUCL6 | I | Os02g0758800 | LOC_Os02g52180 | AK071537 | + | + | + | - | + |
| OsUCL7 | IV | Os03g0259100 | LOC_Os03g15340 | AK107381 | + | - | - | - | + |
| OsUCL8 | IV | Os03g0709100 | LOC_Os03g50140 | AK061596 | + | - | - | - | + |
| OsUCL9 | IV | Os03g0709300 | LOC_Os03g50160 | AK100153 | + | - | - | - | + |
| OsUCL10 | IV | Os03g0791300 | LOC_Os03g57730 | NA | + | - | - | - | + |
| OsUCL11 | I | Os03g0791366 | LOC_Os03g57740 | NA | + | + | + | + | + |
| OsUCL12 | IV | Os03g0850900 | LOC_Os03g63390 | AK062526 | + | - | - | - | + |
| OsUCL13 | V | Os04g0320800 | LOC_Os04g25454 | NA | - | - | - | - | + |
| OsUCL14 | I | Os04g0545400 | LOC_Os04g46120 | NA | + | + | + | + | + |
| OsUCL15 | III | Os04g0545600 | LOC_Os04g46130 | AK119259 | + | + | + | - | + |
| OsUCL16 | I | Os06g0218600 | LOC_Os06g11490 | AK106768 | + | + | + | - | + |
| OsUCL17 | IV | Os06g0266400 | LOC_Os06g15600 | NA | + | - | - | - | + |
| OsUCL18 | III | Os06g0718400 | LOC_Os06g50420 | NA | + | + | + | + | + |
| OsUCL19 | IV | Os06g0721800 | LOC_Os06g50650 | AK243644 | + | - | + | + | + |
| OsUCL20 | I | Os07g0105000 | LOC_Os07g01440 | AU162427 | + | + | + | - | + |
| OsUCL21 | I | Os07g0165900 | LOC_Os07g07170 | NA | + | + | + | + | + |
| OsUCL22 | I | Os07g0542900 | LOC_Os07g35860 | AU173380 | + | + | + | + | + |
| OsUCL23 | I | Os08g0137400 | LOC_Os08g04310 | AK066535 | + | + | + | + | + |
| OsUCL24 | I | Os08g0137800 | LOC_Os08g04340 | AK063041 | + | + | + | - | + |
| OsUCL25 | I | Os08g0137900 | LOC_Os08g04350 | NA | + | + | + | - | + |
| OsUCL26 | I | Os08g0138100 | LOC_Os08g04360 | NA | + | + | + | - | + |
| OsUCL27 | I | Os08g0138200 | LOC_Os08g04370 | EE591745 | + | + | + | - | + |
| OsUCL28 | I | Os08g0138400 | NA | NA | + | + | + | + | + |
| OsUCL29 | I | Os08g0482600 | LOC_Os08g37660 | AK063639 | + | + | + | + | + |
| OsUCL30 | I | Os08g0482700 | LOC_Os08g37670 | AK066830 | + | + | + | - | + |
| OsUCL31 | III | Os09g0469300 | LOC_Os09g29390 | AK059930 | + | + | + | - | + |
| OsUCL32 | I | Os09g0541100 | LOC_Os09g36940 | CI646724 | + | + | + | + | + |
| OsUCL33 | I | Os09g0572700 | LOC_Os09g39940 | AK063784 | + | + | + | + | + |
| OsUCL34 | V | Os11g0426400 | LOC_Os11g23930 | BE040849 | - | - | - | - | + |
| OsUCL35 | IV | Os11g0428800 | LOC_Os11g24140 | AK243634 | + | - | - | - | + |
| OsSCL1 | I | Os03g0807500 | LOC_Os03g59280 | AK068398 | + | + | + | + | + |
| OsSCL2 | III | Os04g0629200 | LOC_Os04g53710 | AK243623 | + | + | + | - | + |
| OsSCL3 | IV | Os06g0216700 | LOC_Os06g11310 | AK101397 | + | - | - | + | + |

^a^, Systematic designation given to rice PCs (UCL, uclacyanin-like protein; SCL, stellacyanin-like protein; ENODL, early nodulin-like protein) . OsENODL1 is a β-GlcY-reacted AGP and indicated in bold and underlined [7]. ENOD-like AGPs identified through amino acid biased method in our previous study are indicated in bold, and OsENODL1, 6, 18 were also named as OsELA1, 2, 3, respectively [13].

^b^, PCs are divided into six types according to the structural characteristics of their protein backbones.

^c^ and ^d^, Locus numbers assigned by RGAP (Rice Genome Annotation Project, <http://rice.plantbiology.msu.edu/>) and RAP-DB (Rice Annotation Project Database, <http://rapdb.dna.affrc.go.jp/>), which can be converted by ID converter (<http://rapdb.dna.affrc.go.jp/tools/converter/>).

^e^, Representative full-length cDNA or EST (Expressed Sequence Tag) is found by searching the RAP-DB locus at the UniGene database (<http://www.ncbi.nlm.nih.gov/unigene/>). NA, not available.

^f^, N-terminal signal sequence predicted by SignalP 3.0 (<http://www.cbs.dtu.dk/services/SignalP/>). +, exist; -, not exist.

^g^, GPI anchor signal predicted by BigPI (<http://mendel.imp.ac.at/gpi/plant_server.html>) and PSORT (<http://psort.ims.u-tokyo.ac.jp/form.html>).

^h^, Putative AG glycomodule identified according to the description in “materials and methods”.

^i^, N-glycosylation sites predicted by NetNGlyc 1.0 Server (<http://www.cbs.dtu.dk/services/NetNGlyc/>).

^j^, Conversed amino acid residues involved in copper binding are His, Cys, His and Met/Gln.
