# Supplementary material for: The Phytocyanin Gene Family in Rice (Oryza sativa L.): Genome-Wide Identification, Classification and Transcriptional Analysis
Source: PLoS One. 2011 Oct 3;6(10):e25184. doi: 10.1371/journal.pone.0025184 (PMC3184959; doi:10.1371/journal.pone.0025184)
Supplement: Table S2 — Protein backbones of rice and Arabidopsis PCs. (DOC) [file pone.0025184.s005.doc]

**Table S1. Protein backbones of OsPCs and AtPCs**

| Name | Type | Protein backbones^a^ |
| --- | --- | --- |
| OsENODL1 | I | MEASRRWPYAAWFMAVLGLVAVFSSSEAYVFYAGGRDGWVVDPAESFNYWAERNRFQV**N**DTIVFLHDDEVGGSVLQVTEGDFDTCSTGNPVQRLEDVAAGRSVFRFDRSGPFFFISGDEDRCQKGQKLYIIVMAVRPTKPSEAPEPAGAAGPVSSKSWSWQAFPPAGATTPPPLPPSWGSAPEHAQAPGKSSLGGSGGGEMSRSSSLGAPPPTSGAAGLAGVVASVVVGVLGALLMF |
| OsENODL2 | I | MTRRVAGVAAAAAAVAAAVVVLAAASCCEARDFYVGGRDGWTTNPAEPYNRWAERNRFQVNDRLGTYADSPEYSGDRSRRRRACSFVCSCRSTLSEFFVKFGAFTVIAVFRYNKEDSVVVVSQGHYDGC**N**ATDPLLRDAGGDSTFVFDSSGPFFFISGDPARCQAGERLIVVVLAVRG**N**ATATPTTPSPPPPPTVPAAPTPRPSPPPPAAGT**N**GTARAPSPPVPAPAPAGSPPPPPPPPAGG**N**FTAPSPAGGM**N**FTAPAPGT**N**GTAAPPPRPSSAPSVRGGALLMLLVVATAGAMALV |
| OsENODL3 | I | MAPPMMATAARPRDDQPRRRQALLLAASALLFLLCGGGAPGAGGVVVAVAATASATATPGLVFHVGGPRGWRVPDA**N**TSYTWWAMNNRFHVGDSLYFRYGGGDSVLVVDREAFDGC**N**ATEPVARFAGGATTVPLGRPGFFCFISGAPGHCDGGQRLIVRVMVHPAPGAPAPAPSAAAAATSHPGASASGPGASSGAAAVAAGGAGAAVAAAAMGVLAGLVLLLQ |
| OsENODL4 | I | MHRQRRSISSPGGNLSPPLLVVAAAVFVVAGLSAAVPGAAAYK**N**YTVGDDKGWYDGLTHPGVDYQEWADGKNFSLGDFLIFNTDKNHSVVQTR**N**ETLYKSCDYNDSGPDDTVEWSAAAPEFSKDAVTVAVPLLKEGSSYFFSGNYDGEQCESGQRFAIAVAHGQGLPPDLRPPAAEAPGPSSSAGAAADAPPTFDFSHPK**N**VSNSPADTSTTAPLDDADDAPTTGGAGRSIARLGSGLAAAATLLFFVVQV |
| OsENODL5 | I | MAHGRVQRMQCWVVVACVVASVSTTASAFVFKAGGTGEWRVPDQQASG**N**VSAYNQWAEHTRFRVGDAIAFSYQPG**N**DSVLLVDKSSYDACNTNTPIDTFADGNTVFTFTRSGPYYFISGNKDNCNRNEKLIVVVMGERAA**N**GTAPAPALAPSAGTTSPNSPPSPPPPPSGIEISPTPEQSINAAARPRAAGIAGAAGLAIGTLFYALV |
| OsENODL6 | I | MVKGTSGYSYGLGLACFALVVAMAGATQLKVGGGNGWSVPAANAESYNDWAEKMRFQIGDTLVFVYPKDKDSVLVVEPADYNAC**N**TSSFDQKFADGNTVFTLDRAGAFFFISGVDANCRAGEKLIVMVLASR**N**GTATATAPSPPPASSTAPPPTSPAPASPPPSSPSPPPASPPSPSSSGAAAPTTPPPASSPPSTPTPASPAPSASSPPAPPSANAPSAQGAR**N**PSATSSPPPAANGAAHAAVAASGLAAGIIGYAMLAL |
| OsENODL7 | I | MASSCSVLVVACSFAVLHVVAIAGATQYKVGGDGGWGVPGAGDEPYNTWAEKTSFQVGDQLLFVYPKDKDSVLVVEPADYNACNTASYDSKFADGNTAVTLDRAGAFFFISGVDANCRAGEKLIVMVANATGSSASPPSSSSSPSSPSGGGGGGGAPAGQAPPGAPATPAGTNSSPANGGAAGGGAKSGAGLTVAASGLAGSLIAAIACVAIAI |
| OsENODL8 | I | MAIVSVTTATAAAGVLILVVAAGAAAAAGARRHHVVGGDPGWAVASDVLAWSADRLFTVGDTLWFAYSAEDGGVAEVGGEEEFESCDAGSPVRMYTEGLSRVDLGGEGSRYFVSADPDKCGGGLKLRVDVRAPVAGTTPPPGSSRKGDRAAAPAPAPLASSGGRGVATSRTCVMLCCLLFLAI |
| OsENODL9 | I | MASLAIFAAVAFVLLAASVSSSSAALYTVGDARGWAVPPTGSESYNHWGLKNRFRVGDVVEFKYV**N**ESVVVVNHEGYR**N**CSSLSPVIRFTDGDTKYLLDRPGLVFFISGVQERCERGLRMRLRVRPAAPGPAQAPAPGPTRAALTLRRPPIGAPRPAAVTAAFTPTSPSASRPSARTSPSPSPGPAQAPSGASGRALTGFSMAAALLVVCVVSVFILV |
| OsENODL10 | I | MRGASALASLVAAAAVALLLLIDGCGGAMYKVGDLDAWGIPPPSKPDVYSRWAKSIHFALGDSIWFLYPPSQDSVVQVTPVAFAACQASDPVLKLDDGNSVF**N**LTTPGRVYYISAALGHCRKGQRLAVDVPMANGTYLPPTANDLAAFAPMPAEAPAGFESAALGPAGARQSAAPRAAAAGGAGSVLLAALAFAVFLL |
| OsENODL11 | IV | MEGACLRLVVNPQQPTTLLAVFLLLLVAAAVAPPHVLAADHVVGGSIWSIPPRPGLYRAWAD**N**RTFVAGDNLVFRFETGMYNVVQVGRREFDDCTADDPYRDWTDGPAVVTLGSAAVRYFICTVGNYCSLGVKVYVASQNAP |
| OsENODL12 | III | MVMTRRRAALLVVAMCACAALPSTTTANKFSINWKPNT**N**YSDWPAQHGPFYKGDWLVFYYTAGQADVIQVDAAGYNTCDATNAIS**N**YSKGRTYAFEL**N**ETKTYYFICSYGYCFGGMRLQIKTEKLPPPSPPAAAKDKSAAAFTASRASLFYAAAAAVLAAILRMF |
| OsENODL13 | IV | MALARGGRGGDKAICSVHLGLLLLLVILALQCGVEPAAARREWPVGDGAGWSPGVVGWPNYKPFKAGDVLVFSYDASAHNVVVVGDVDYALCRAPANATAYGSGDDRVALPPGVTFFVSGFPGDCDKGMMKIAVTAR |
| OsENODL14 | I | MTTTNHLVSPALLLLLALLGASVRRAGATTFEVGGEHGWAVPPAKDAGVYNDWASKNRFLVGDSVHFKYAKDSVMVVTEDDYNKCKAEHPIFFSNNGDTEVGLDRQGLFYFISGVAGHCERGQRMVIKVIGHDAPPPASPPPPPSNAPPTPPHPSGAASALGAGGLAVAAMLLPVFVYGV |
| OsENODL15 | III | MASRTQYAFLLLSAFMASLFAGSAAGVYHIIGAGKGWRMAP**N**KTYYADWARTR**N**ISVGDKLMFLYRSGVYNIVEVPTKELFDACSMR**N**ITNRWQNGPTIIELTQPGPRYYFCGVGKHCEEGEKVAI**N**VSVSAPTLPDSDADADDDDADDSDSSAATPATAADLLIYLAGLAACLLPALLLI |
| OsENODL16 | I | MAGAALLFPATVIAAACVVLSGGASAAPPGRVFVVGGDGPRGWSQPTGTDETYNHWASRNRFHIGDFLDFKYAK**N**DSVVVVSRADYKLCSADKPVQRFDDGADVRFRLDRNGNFYFISGAPGHCKAGQRMTVRVMADHAAKGAGGGDSPAGAPSPDGDGDDEDDSGGSYRTPGYGYSSGSPPTPPHG**N**TSAAAVVSPSRGGGGGGGYHRVAGVAAAALLVLA |
| OsENODL17 | I | MARRDQLVSFLCFFLIVSAVAGGLCVSATVLPMRVGKQYVVGGRSGWRTPPPASVDLYAKWAAGIRFYVADSIEFVYK**N**DSVVKVDKFGYYHC**N**ATAAAANDGSVLFLLDAPGFAYFSSADADHCKKGQRLMINVDSAPSPSPSPSPAPQEAATASAATSSSAATAAHALLLAAMAMMGLILGEW |
| OsENODL18 | I | MAGAVATVSVGLAWLGLMAAAASATQFRVGGGRGWSVPDANAEPYNSWAGRMRFQIGDQLLFVYPKEMDAVVVVDQGAYDAC**N**TSSSVAGGGGGRYDDGNTVFTFDRSGPFFFISGNEANCRAGEKLVVVVMADRGGRHAPPPSPPAVPPPVAPVPMPSPASSPPSPAPAAATPSLAPSPVATTPSPSPSVSPMAPAPAPTTSTPSSPPAPAAMAPSPSTTPGGVAQPPPPPGTDGANATTPAAPAANDRSGAAAAAPVVAGVVVTSLGAYIGYAMLAI |
| OsENODL19 | III | MVCHRMLPLLVVAVALLPAAAVAT**N**YTVGDEKGWNPDVDYTAWVKKHRPFYKGDWLLFEYQNGRSDVVQVDEVGYDNCDKANAISSYSKGHSYAFQLKEAKDYYFICSYGYCYKGMKLAVTAKKGSASSSSSGSGDSSSSSKSDTASSKSKSSAAASSLANPSYAALLAVAIIFLRML |
| OsENODL20 | I | MAKVLATVLCAALAFAAAVAVANARDLVVGGNNGGGWKVPAQPDALNRWAEATRFHIGDNLVFKFDGAADAVLEVTRDDYNHCGTGSPVATHKPTGGAATVPLTSSGYHFFVGAAPGSCDKGERVIVLVMSEKHSRRGQGFFAPVPAPAQSPLAAGLFQAPAPAPATGNAGRTAASGAVLVAAALLGAAVAGF |
| OsENODL21 | III | MACHLLLVAVVAGFAVSLAGATDHIVGANHGWNPNIDYSLWSG**N**QTFYVGDLISFRYQKGTHNVFEV**N**QTGYD**N**CTMAGVAG**N**WTSGKDFIPL**N**DSRRYYFICGNGFCQAGMKVAITVHPLKH**N**ATGDGAKNHGGDGAAQEAAAAAMPGAAVWMAVLAVAAAAVAILP |
| OsENODL22 | I | MTYIVITMSIVVAVQLAAVFSMASATPAPS**N**ATATTSHGR**N**TTAPPPPFGA**N**HTVGEGAGWFFDGNA**N**ASVA**N**YSAWAA**N**RTFYLGDYLSFSTNTDNTVVHTT**N**ATVYKLCGDGGAAAAAGCSGGGWKTEEAFLTVMLTAEGANYFFSDAGGGEHCRKGMRFELAVARGRGLPPVPASYYEPLSAAPPAAGCSSSMVVALAAGVAIAAILVL |
| OsENODL23 | I | MAAAATVFVGAASGASYTVGEPGGGWDTQT**N**LTAWASTVDLRRGDQLVFRYDASAYDVVEVTRAGYLSCSAASPVSAALRTGNDVVRLDSAAGWRYFIYGVEGRCAAGMKLQVRVTDAGAGCNNTLPSPSLAPAPPGAPSPGITICSGGPPTVIMTPGVISYGAASRSSA**N**LSSSLLVAMVSLLLGIIVV |
| OsENODL24 | I | MSRSTAATAAVVVAVAVVAAMAMPAAGQGAPSGSPAPPYK**N**HTVAGADGWFF**N**ATS**N**TTSG**N**YSDWAAGETFYLGDYLIFKTDDSSSVVQTS**N**ATAYSLCDAEGPETLIYSPGHGDAASASPRAATIAVPLTVEGANYFFSEAGDGAQCEEGMRFEIKVAHGRGLPPDLAHPPPPPKPRVLAPPPDGTSMSPGVAGAGAGAAGDLTEGKSGGSRAGVGLLGVAVGVGLAVLVAA |
| OsUCL1 | I | MVDMKAAICIAAAVSLIHVVSAADYTIGSAAGGWGGEYKAWVASQTFSPGDTLTFKYSSYHNVVEVTKDDYEACSATSPVSADSSGSTTIVLTTPGKRYFICGAPGHCQSGMKLVVDVADRPAPATPSPPPLLPPSPRHKRRTAPAPMPLPPAQAPVWSPAPAPAATQRRHSGHKKHRSRHLPPKPAPAMAPTVQSVEADFPAAAFAPMSSPPPPPPMSSDASAVVRQKWSDVIVGLVALGLVVLGV |
| OsUCL2 | IV | MARGSAAAPGLLLALCCAAAAIAVVHGEDWAVGDNKGWSFGVAGWENGKRIQPGDELVFKYDAKIHNVVEVDRAGYGGCTVTGPSKVYNSGDDRIKLAGGEAFFICSIRDHCTAGMKVKVAVTANA |
| OsUCL3 | IV | MARAGSVCVAVLLAVCCAETILVAGATEWHVGDDKGWTFGVAGWENGKAFKVGDVLVFKYSPMMHNVLQVDHAGYDGCKVGAGDKKYASGNDRITLAAGKVFFICGFPGHCANGMKIAVATK |
| OsUCL4 | I | MAGVHGLAAAGLVVLLLAAVAPAFAVDYTVGDTSGWSSGVDYDTWAKSKTFSVGDSLVFQYSMMHTVAEVSSADYSACSASNSIQSYSDQNTKIALTKPGTRYFICGTSGHCSGGMKLAVTVSAAAATTPTPTASSSPPSTATPATPSSDPGMDTPSSTPDATTTPTTTTTKSTGSTGGASGSEARSVMGLLVGAVGLAMMG |
| OsUCL5 | IV | MAARGRGSACNGAAVLGAAAAVVIVGFLVMSAAPLAEAARYTVGDSSGWRFYAEGWAKGKTFRAGDVLEFKYNAVVHDVAAVDLAAYRSCTVPKGVRKMRSGRDKVTLRKGTHYFICTEPGHCKAGMKLAVRAI |
| OsUCL6 | I | MASCGASCLTIAALLLAACASSAAATSYTVGDASGWTIGVDYTSWAGSKSFKVGDSLVFKYASGAHTVVEVSAAGYLACAAANALGSDSSGSTTVALKTPGKHYFICTIAGHCAGGMKMEVDVSGSSSSSSGGGGGGGGGGSTPSSPSSPTPTTPNPSTPTPTTPYPSTPMPTTPYPSTPMTTPTTPYTTPTSPACSGGAGATPVTPVTPGTVPFMSYNGAGGLGPVALATIGMVCFVVFVQLGLL |
| OsUCL7 | IV | MAQGRGSAAQGLALGLLLVCLLVGSDVAAAATYNVDWSFGADSWSKGKNFRAGDVLVFSYDPSVHNVVAVDAGGYSGCRESGTKYSSGNDRITLGRGTSYFICSFSGHCGAGMKMAVTAS |
| OsUCL8 | IV | MARGRGSAMRGAVAVAFLAVVVSCIFLSGCGVADAATYYVGDSLGWSLGSGSWPSGKKFHAGDILVFRYLPWMHNVVAVDEDGYADCNPPPFSRYYTSGSDSVRLARGDNFFVCTRYGHCNLGMKMVVTAV |
| OsUCL9 | IV | MAQGRGSAAGRRNAAVLAMVLLCVLLHGELAESAVYTVGDRGGWGFNSGGWLRGKRFRAGDVLVFKYSPSAHNVVAVNAAGYKSCSAPRGAKVYKSGSDRVTLARGTNYFICSFPGHCQAGMKIAVTAA |
| OsUCL10 | IV | MASRRITLELAAVVVVVAAAVAGSLPATTASATAYRVGDDSGWDNGVDYDAWAHGKRFKVGDTLDVEFLYAEGAHNVVVVEDEGSFEACVAPANAPTLSSGDDTVALNQAGRWLFICSFDGHCQSGMKLAVAVTH |
| OsUCL11 | I | MASKQMLAVAAAAVALAVLLPARGAAATEHMVGDGNGWILGFDYAAWAATKQFRVGDTLVFRYKGT**N**HTVVEVGGADFKAC**N**KTASANEWSSGEDRVALDKEGRRWFFCGVGDHCAKNMKLKITVIAAGAPAPGASEAPPPPSSAAGKARARVAHAAAAAAVTAAAAAMLAL |
| OsUCL12 | IV | MAGRGSAMAAVGVLVLLCVLLHGEMAESAVFTVGDRGGWGMGAGSWANGKRFKAGDVLVFKYDSSAHNVVAVNAAGYKGCTAAPRGAKVYKSGNDRVTLARGTNYFICNFPGHCQAGMKIAVTAA |
| OsUCL13 | IV | MELTIQTCGFACACAVFNYQAGVHNVVAASAAEYRSCRVRNAADAAATAAGSAEVELKEGVNYFICGVPGHCAAGMKLRVVADEFPSADTK |
| OsUCL14 | I | MAQSCLALAVCVLLVHGGAARVAEAASYNVGNSAGWDISADFPSWLDGKSFFVGDTLVFQYSKYHTLSEVDEAGYR**N**CSTASAVLSSSDG**N**TTVALTAPGDRYFVCGNELHCLGGMRLHVPVSEPASPGGAGATPASPGGGGALSPGAAGDAGVPTLDLGGSHRVTVGPAVATWLCIIAAALFVW |
| OsUCL15 | III | MQKHTGTSRQMAGLLPGVLVAVLLAAAAAPASAKDYTVGDSSGWTTGVDYTAWARGKTFNIGDTLLFQYTSAGHSVVEVSEADHTSCSAANPLRSYKDGTTIVTLTRSGTRYFICGSTGHCGAGMKLTVTVATLSGSAAGGTRLAKPSSSDADPTTTTTTRTSSATGGATGSWAPRTATWLLFFAAVGALL |
| OsUCL16 | I | MASSLALVALLLVSCAVVAAAATKYTVGDTSGWAMGADYTTWASDKKFKMGDTLVFNYAGGAHSVDEVSAADYAACTASNALQSDSSGTTTVTLKTAGKHYFICGIAGHCSNGMKLVVDVAAASPAPAPKAPSTTPTTPSTTPATPASPGTSSGLTPTTPATVLAPPAKQSAGAAGLRARSWAMLGLAGLAAVQLGLF |
| OsUCL17 | IV | MAVAAARRGRGSASGGGGVVLLCLVAAALLMEAVPAAEAGGKTYYVGDAAGWGRNLDWWLAGKTFYAGDVLVFKYNKEYHDVAVVGGKGYRRCKVPRNKDTAVLRTGYDQVTLRRGNNYFICGMPGHCDAGMKLAVKALCFLYCTGSLMI |
| OsUCL18 | III | MPPSPPLQLVALLLLSLLLRSATAAEYTVGDGPWDTGTNYATWSDKHAFLAGDILVFQYVRSQHNVLQVTEATYRSCDTGGGGVAGVIKSYDTGYDRVQLTEP**N**ATYWFICDFPGHCLGGMRLAVKVAAAAAGGGGGGGSPPPSGVPLHPPAAGGAGRSQWPAWGLTLAVLLVVFHYCIIIF |
| OsUCL19 | IV | MPPSPPLQLVALLLLSLLLRSATAAEYTVGDGPWDTGTNYATWSDKHAFLAGDILVFQYVRSQHNVLQVTEATYRSCDTGGGGVAGVIKSYDTGYDRVQLTEP**N**ATYWFICDFPGHCLGGMRLAVKVAAAAAGGGGGGEGARRPAASPCIRRPPAARGGANGRRGG |
| OsUCL20 | I | MVTKKQQAALLAVVAVAALAQVAAAAVHPVGGNGAWDTTGNYNAWSVSQKFSQGDSILFTYPSSHDVVEVPKASYDACSPANALASYTGGSTTVKLDAPGKHYFICGVPGHCAAGMKLEVTVAAATATKPRHKKGAAPAAAPAMPPAVSSPTEEMPAVTSPTGSPAPSSASAASTIAINVAATLAAGMALAFLAM |
| OsUCL21 | I | MASRQVLLLAIVSAVALLPAMVSATDYTVGDGHGWTLEYPSTNWADGKSFQIGDKLVFTYTKGKHTVTEVDGAAFHACNRQGNTLMTWNSG**N**DTVALDKAGKRWFFCNVDNHCELGMKLVVDVADPNAPAPASPPPPPSSSSSAGRLNYRARGGAVAGAVAAAALVWF |
| OsUCL22 | I | MKASSVALLAAVMAVAAVASTAVAKDYTVGGSYGWDTYVDYDKWAAGKTFIVGDTITFKYEPYHNVVEVPAETDYDGCVSTNPVSVHSGG**N**TTFELAAAGTRYFICSIPRHCL**N**GTMHVKVTTVPYSASAAAAAAADAGPSPAPLPSPPADEQQHRSNSASSPAAGPSSSAASTPRHRKQPAVAVAGLALAALVAMAA |
| OsUCL23 | I | MEWARGPAMAAAAAAAVVMVAAVLAGQAMAAGATTYTVGAPDGLWDMETDYKEWVARRTFHPGDKLTFTYSRELHDVVEVTKAGYDACSNAN**N**ISAFRSGNDLVALTAVGTRYFLCGLTGHCGSGMKIRIDVVAAASSGPAAAAAPLPSTSSVTAAVAGSRLVLVLLYALLPLW |
| OsUCL24 | I | MAIMAARALLVVAMAAAVLGTALGATYTVGAPSGSWDLRTNYDQWVSNINFRAGDQIVFKYSPAAHDVVEVNKADYDSCSSSSPIATFNSGDDTIPLTATGTRYFICGFNGHCTGGMKVAVKVEAATGSNPAPSPMTPRPRTPTAMAPNAMPPTAGGRPVPPSNSASQPAGVASLVGLSLGAIVVGLMAF |
| OsUCL25 | I | MAARALLVVAMAAAVLGTAMGVTTYTVGAPAGSWDTRTNYAQWVSAITFRVGDQLVFKYSPAAHDVVEVNKADYDSCSSSSPISTFNSGDDTIPLAAIGTRYFICGFPGHCTAGMKVAVKVEAATGSNPTPSPLAPLPRTPTVMAPNAMPPTNGGRPTPPSSSASKPVGVASLVGLSLSAIVAGLMVF |
| OsUCL26 | I | MAAAVLTTTATGATTYTVGAPAGSWDTRTNYAQWVSAVTFRVGDQLVFKYSPAAHDVVEVTKAGYDSCSSSGPVATFNSGDDTVPLTATGTRYFMCGFPGHCAAGMKIAVKVEAATATGGSGTALSPMAPRPRTPTAMAPNAMPPMAGGRPVSPSSSASKSTGVASLVGLSLGAIVAGLMSF |
| OsUCL27 | I | MAASARALLVVAVAAAAAVLATTAMGATTYTVGAPAGSWDTRTNYAQWASAATFRAGDRLVFRYSPAAHDVVEVTKAGYDACSAASPIATFNSGDDTVPLAAVGTRYFICGFPGHCAAGMKLAVKVEAAAAAPGGSSTTPSPSPSPAALPPVNGGRPVTPSSSASKSGGVVESLVGLGVGAMAAGLMVFY |
| OsUCL28 | I | MALLAVVVVAAAAFSTASGASYGVGKPNGGWDLQT**N**YTSWASSITFRLDDKLVFKYSAAAHDVVEVTKDGYLSCSASSPIAVHRTGEDAVELGRLGRRYFICGVPGHCDAGMKLEVRTLCSIPSPPPPGSDGDGNGTPGGICIDGSSPPTIISTPGVVSYGSAPGSSGSATTALAIMAAATVMLLSLIIV |
| OsUCL29 | I | MASPSALIAMLLVMVVGCAAVASAMELSFIVGDAQGWNTGVDYTAWAKGKTFEA**N**DTLVFRYARKQHTVTEVTKSDYDACTVSGKPISDFEGGALVTFIALSPGEHYFICKIGNHCASGMKLAVTVSNSSDTPRPQPWIGPYSTPASASAHLHAGGAVVAAAVGILLNLALF |
| OsUCL30 | I | MASSSVLIKLLVVVGCAAAASAATLTVGGSSGWTLGQNYDTWASGQTFAVGDKLVFSFVGAHTVTEVNKNDYDNCAVASNSISSTSTSPATLDLAAAGMHYYICTISGHCAGGMKLAINVGSGSGSGSGSGTPPSTTPGSSGTPPATPSSPSKPTGGASAGLQASAAVAAAAGVLVKLALF |
| OsUCL31 | III | MAVSSAVLVGLLVVSCAAVAAATRYTVGDGEGWTTGVNYNNWANGKFFRQGDELVFNYQARAHTVTEVSQTNFDSCNGNSPLSNDNGGSTTIRLSYPGMHYFICTIPGHCSSGMKLAVNVNGDPSYSAASSPAAASAVAAAAAGALIKLALF |
| OsUCL32 | I | MVAKAACLPSAAAVALLLLAAAAAAGFAGATEYTVGDSEGWTIGPSYLAWSTKY**N**FTAGDTLGQFVRSLAVDSLMISISSTSGSIRSPDVVASCAVFSYVQRQHDVLRVSQDAFRTCDPA**N**QTVQRWASGRDVVELAAPGSYYFIC**N**VSGHCLGGMKFSVAVGEPLPPPSPPPPPPRAPFLAPPPPPPVGSGAAAASSTWRRRRVALMVQVSCLALIIIGMWN |
| OsUCL33 | I | MGSIGGVAVVLVGMAAMLVGMASAATYNVGEPGGAWDLTT**N**YTNWVAQKRFHPGDQIVFKYSAQRHDVVEVNKAGYDSCSTSTSIATHTTGNDVIPLTSTGTRYFICGFPGHCTTTGTGNMKIQIDVVQADSSSAPAPVATTTPPSPPSSAATSLKATAAAAVLLAALLIMA |
| OsUCL34 | V | MSWETPRDGASPSRMTAGPVGRLSPPATHLAGVHNVVAASAAEYRSCKVRNSADAAATAAGSAKLDLKKGVNYFICGVPGHCATGMKLRVVAN |
| OsUCL35 | IV | MGKYSVGLVVLGLLALAFSTTVLAETHVVGDSNGWDFSVSFDSWADGKVFAAGDTLVFNYKPGAHNVLAVDAATYRSCKVGSSADSVAAATGTASFLLKKGVNYYICGVPGHCAAGMKLRVVAN |
| OsSCL1 | I | MAMAMNSVLVLMLGLAMAATSSAAVYKVGDTSGWTILGNV**N**YTDWAVKKTFHVGDTIEFKYPQGIHNVVEVKKADYDSCTNSSPIATHTSGDDKIAIKAAGHRFFICGVPGHCAAGQKVNIRVLKPQRSSSSDAPSPAPAASKRGAAAAPSPAVSSSPPESSSPTTDSSSSSTTTAPAPNASAAAGGGGAKAAFAAVALALVAATAMLQ |
| OsSCL2 | III | MSSSSSSLKALVAFMAVATVAELAAGSKTWAIKWASGGNYGDWSSKNTVAVGDSVVFTYGTPHTVDELSAADYTACSFAAPLSSDAGGSTTVVFDKPGTRYFACSSGSHCSMGQKVAITVSNSTAPPSSSKGGSSSYGAAAGGGAELASKLVVGLAVGAGAILAL |
| OsSCL3 | IV | MEGRISSATLVVAAVLAMLVLVPAAARAERFVVGDAARWTWGY**N**YTDWVIKKGPFFQ**N**DSLVFMYDPP**N**ATTHAHSVYMMRNAADYQSCNLKAAKLVANVMQGAGSGYEFVLRKRKPHYFVCGERGGIHCTMGQMKFIVKPKSSACRDD |
| AtENODL1 | I | MSAIMKSLCFSFLILASFATFFSVADAWRFNVGGNGAWVTNPQENYNTWAERNRFQV**N**DSLYFKYAKGSDSVQQVMKADFDGCNVRNPIKNFENGESVVTLDRSGAFYFISGNQDHCQKGQKLIVVVLAVRNQPSAPAHSPVPSVSPTQPPKSHSPVSPVAPASAPSKSQPPRSSVSPAQPPKSSSPISHTPALSPSHATSHSPATPSPSPKSPSPVSHSPSHSPAHTPSHSPAHTPSHSPAHAPSHSPAHAPSHSPAHAPSHSPAHSPSHSPATPKSPSPSSSPAQSPATPSPMTPQSPSPVSSPSPDQSAAPSDQSTPLAPSPSETTPTAD**N**ITAPAPSPRTNSASGLAVTSVMSTLFSATFTFLMFA |
| AtENODL2 | I | MTFLKMKSLSFFFTILLSLSTLFTISNARKFNVGGSGAWVTNPPENYESWSGKNRFLVHDTLYFSYAKGADSVLEVNKADYDACNTKNPIKRVDDGDSEISLDRYGPFYFISGNEDNCKKGQKLNVVVISARIPSTAQSPHAAAPGSSTPGSMTPPGGAHSPKSSSPVSPTTSPPGSTTPPGGAHSPKSSSAVSPATSPPGSMAPKSGSPVSPTTSPPAPPKSTSPVSPSSAPMTSPPAPMAPKSSSTIPPSSAPMTSPPGSMAPKSSSPVSNSPTVSPSLAPGGSTSSSPSDSPSGSAMGPSGDGPSAAGDISTPAGAPGQKKSSANGMTVMSITTVLSLVLTIFLSA |
| AtENODL3 | I | MGLVMRFDLYLMFVMLMGLGFTISNGYKFYVGGKDGWVPTPSEDYSHWSHRNRFQV**N**DTLHFKYAKGKDSVLEVTEQEYNTC**N**TTHPLTSLSDGDSLFLLSHSGSYFFISGNSQNCLKGQKLAVKVLSTVHHSHSPRHTSPSPSPVHQELSSPGPSPGVEPSSDSNSRVPAPGPATAPNSAGLVGPGMVVLVIMISSLF |
| AtENODL4 | I | MVFVKMTDVYLMIVMLMGLGFSIELSNGHKFYVGGRDGWVLTPSEDYSHWSHRNRFQV**N**DTLYFKYVKGKDSVLEVSEKEYNTC**N**TTHPLTSLSDGDSLFLLSRSDPFFFVSGNSGSCLKGQKLAVTVMSTGHHSHTPRHPSPSPSPSASPVRKALLSPAPIPVHKALSSPAPTPGVDPSHSEVLAPAPGPAAAVRNLAGSVAPGVISLGLVLVIMISSMV |
| AtENODL5 | I | MDSSKKIIIVMFLVTFYMFSCVSSTEFEVGGENGWIVPKSKTLGDAFNQWASDNRFKVGDTLRFKYTKDSVLVVSEEEYKKCKATKPQLYSNNEDTVFKLDRPGLFYFISGVSGHCEKGQKMIVKVMETESSTESPPPSSSSSSSSSSSLPASTPKAKKSNAFKTAVQFSSSGFVVSAVLIVSVFGLV |
| AtENODL6 | I | MGGQKIVLLSIFVCFYVFSLVSCTEFEAGGENGWIIPQSS**N**QSDIFNQWASKNRFKVGDTIRFKYKKDSVLVVTEDEYKKCQTTKPELYSNHDDTVFKLDRPGLFYFISGVSGHCEQGQKMIIKVMEVESTPQSPPPSSSLPASAHKKNHAVRKTSRFLGAGLVTISILVITVFSLV |
| AtENODL7 | I | MMMMMMRSTC**N**LTLMLCICALVVASMAAEGPRDFKVGDEFGWRVPLQ**N**DSAVYSHWASSNRFHIGDSLSFVYDKDSVMEVDKWGFYHC**N**GSDPITAFDNG**N**STFDLDRPGLFYFISGSNQHCTSGQRLIVEVMHIHQHHDHDASMPPSMSPLSNSASPYASASASSAASSLPTACLLIPLFLTIASFRFISY |
| AtENODL8 | II | MGVMSLSKTMVVVVLQVMILLGQEIGKVSSTLYKVGDLDAWGIPIDAKVYSKWPKSHSFKIGDSLLFLYPPSEDSLIQVTPSNFKSCNTKDPILYMNDGNSLF**N**LTQ**N**GTLYFTSANPGHCTKYQKLLVSVGTYSAEAEALSPSSAADAPSYQNAFGSIPLSQKSSASSSLISAFSTVAASLACAVVGAIM |
| AtENODL9 | I | MARNLKSMMLCGFGLLCFLMIVDRAYAREFTVGGATGWTVPSGSQVYSQWAEQSRFQIGDSLLFVYQSNQDSVLQVTRDAYDSCNTDSPTAKFADGKTSVTLNHSGPYYFISGNKDNCKKNEKLVVIVMADRSGNKNTASSPPSPAPAPSGESAPSPPVSGTFEMTPAPTPTTSEDTPNSAASSLSFVAALLGAALASTLFLH |
| AtENODL10 | I | MSSVMMCCCLLLLFGLLSEGREILVGGKSNTWKAPESRDETLNQWSGRTRFKIGDSLLWKYNAE**N**DSVLQVRQTDYERCDRSEPIRGYKDGHTNIELKRSGPFYFISGEEGHCQRGEKLRVVVLSPNH**N**RSVVDAPAPVNIVLSPNY**N**RSVAAAPLNAHIMNKGSLNTAWSLLLLLPLGLLV |
| AtENODL11 | I | MVSLISIVSVVFLLFTTFYHFGEARIINVGGSLDAWKVPESP**N**HSLNHWAESVRFQVGDALCSFVMMVKIRMLVIVGYTFMFKYDSKIDSVLQVTKENYEKCNTQKPLEEHKDGYTTVKLDVSGPYYFISGAPSGNCAKGEKVTVVVQSPNHPKPGPAAVTPTLPPKPSTTPAAPAPAPPTPSPKSSTSTMAPAPAPAKSSAVGLVAGNGIFWASTLVAVIGLAFA |
| AtENODL12 | I | MGIIVPVLTLVFLLFAKVSHGASNPRVILVGGSVGSWKVPDSP**N**NTLNHWAENNRFKVGDFIVWKYDMKVDSVLQVTKEDYESCNTANPLKQYNDGNTKVALDKSGPYFFISGAPGNCAKGEKITLVVLAERKSGGGSSSGDAPKVSPVSPTAQTPAPAPGPAAAHNAAVGLKVASGWFLTAVVVGLAMA |
| AtENODL13 | I | MAQRTLVATFFLIFFLLTNLVCSKEIIVGGKTSSWKIPSSPSESLNKWAESLRFRVGDTLVWKYDEEKDSVLQVTKDAYINC**N**TTNPAA**N**YSNGDTKVKLERSGPYFFISGSKSNCVEGEKLHIVVMSSRGGHTGGFFTGSSPSPAPSPALLGAPTVAPASGGSASSLTRQVGVLGFVGLLAIVLL |
| AtENODL14 | I | MFLSASMASSSLHVAIFSLIFLFSLAAANEVTVGGKSGDWKIPPSSSYSFTEWAQKARFKVGDFIVFRYESGKDSVLEVTKEAYNSC**N**TTNPLA**N**YTDGETKVKLDRSGPFYFISGANGHCEKGQKLSLVVISPRHSVISPAPSPVEFEDGPALAPAPISGSVRLGGCYVVLGLVLGLCAWF |
| AtENODL15 | I | MASSSLLVTIFLCISVFFFSSVNANEVTVGGKSGDWKIPPSSSFSFNEWAQKARFKVGDFIVFKYEAGKDSVLQVTREAYEKC**N**TTSPKASYTDGNTKVKLDQAGPVYFVSGTEGHCQKGQKLRLVVITPRNSAFSPGPSPSEFDGPAVAPTSGAAKLAGGFSVVFGLVLGLWAFFF |
| AtENODL16 | I | MARVAVLVAGAVLAFLLAAT**N**VTAKRWTVGDNKFWNPNI**N**YTIWAQDKHFYLDDWLYFVYERNQYNVIEV**N**ETNYISCNPNNPIA**N**WSRGAGRDLVHL**N**VTRHYYLISGNGGGCYGGMKLAVLVEKPPPPPAAAPNKNSARRTFSVSGFAYQFLIPVAVFAAVGTRY |
| AtENODL17 | III | MARFTVLITAVVLAFLMAAPMPGVTAKKYTVGENKFWNPNI**N**YTIWAQGKHFYLGDWLYFVFDRNQHNILEV**N**KTDYEGCIADHPIR**N**WTRGAGRDIVTL**N**QTKHYYLLDGKGGCYGGMKLSVKVEKLPPPPKSAPVKNIGSVSMVTGLAQFMIPVSLFAFPAMWDVISRMW |
| AtENODL18 | I | MSPSCSSCVNVLLIMCLMLLSLSADAYK**N**YTVGESTGWFDIQERPSANYQKWADSKSFSLGDFLIFNTDS**N**HSVVQTYDFKTYKDCDYDNNENNDTTEWSAA**N**PSATSPVPVSISVPLVKEGSNYFFSGNYDGEQCKFGQHFMI**N**VTHGQGLPDSSSPDDAAAPGPSESSQSGDDEVAPDTIVPANFDHPKDIESADDDKEVHSKKSSSSTTKTSLFCFVFMGLFASF |
| AtENODL19 | II | MGRSMVLISAVVLAFLVAAPIPEVTAKKYLVGDKKFWNPNI**N**YTLWAQGKHFYVGDWLYFVFYRDQHNILEVNKADYEKCISNRPIR**N**YTRGAGRDIVPLYETRRYYLLDGRGGCVQGMKLDVLVETPPPPPPFTPPPPAQ |
| AtENODL20 | II | MMGKYLWALVYVTVMILIIVVEVESSLHRVGGGRYTWNSDV**N**FSDWANHQRFYSGDWLYFGF**N**RTRHNILQV**N**KSSYEQCVDNDYIF**N**ITRGGRDVFQLLEPKPYYFICGRGYCLKGMKLAITVLPQPPPSAPT**N**FTSTTTPLIPPNAITAAILIFAFKALLL |
| AtENODL21 | III | MFLWLVIVLTISASVSSYEHKLNWVVPPA**N**SSESFNDWASNKRFQVGDIIQFKYKKDSVMQVTKESYKQC**N**SSHPRFYSNTGKTRFMFDHSVPYYFISGTSGHCEKGQKMIVEVISRDHTTTSAAPPAAFAVLLCFFSLSLYFVA |
| AtENODL22 | IV | MAQSSGHVSYVAVTVPIAIVMTVLCLFLANAVTYARRPTTYIVGGDDGWDPVVPMDTWARGKTFYAGDILEFKYDYQRFNLIVV**N**RTGYETCEANVGAIEYSSGDDKIQLNYGYNYFIGTYTPEDCTTGLKMAIKALAPR |
| AtPC1 | IV | MAKGRGSASWSARAIVTLMAVSVLLLQADYVQAATYTVGDSGIWTFNAVGWPKGKHFRAGDVLVFNYNPRMHNVVKVDSGSYNNCKTPTGAKPYTSGKDRITLSKGQNFFICNFPNHCESDMKIAVTAV |
| AtUC1 | I | MASREMLIIISVLATTLIGLTVATDHTIGGPSGWTVGASLRTWAAGQTFAVGDNLVFSYPAAFHDVVEVTKPEFDSCQAVKPLITFANGNSLVPLTTPGKRYFICGMPGHCSQGMKLEVNVVPTATVAPTAPLPNTVPSLNAPSPSSVLPIQPLLPLNPVPVLSPSSSTPLPSSSLPLIPPLSPALSPATAAGTSLPLFPGSPGSSSSTTSTKTVGTFPSSTTGTTADLAGADSPPADSSSAAKTLVLGFGFMVAMMLHLF |
| AtUC2 | I | MAMNGLSKMAVAAATALLLVLTIVPGAVAVTYTIEWTTGVDYSGWATGKTFRVGDILEFKYGSSHTVDVVDKAGYDGCDASSSTENHSDGDTKIDLKTVGINYFICSTPGHCRTNGGMKLAVNVVAGSAGPPATPTPPSSTPGTPTTPESPPSGGSPTPTTPTPGAGSTSPPPPPKASGASKGVMSYVLVGVSMVLGYGLWM |
| AtUC3 | I | MGSTVAAALLLFLAAVPAVFAATFKVGDISGWTSNLDYTVWLTGKTFRVGDTLEFVYGLSHSVSVVDKAGYDNCDSSGATQNFADGDTKIDLTTVGTMHFLCPTFGHCKNGMKLAVPVLAAAPSPSTPSSPPSTPSTPSSPPSTPSTPSSPPSPPSPPSPSLPPSSLPPSASPPTNGTPDSETLTPPPAPLPPSLSPNAASKGVMSYGIIGVTMILMYAVMT |
| AtUC4 | I | MSTLLGCLVLIFSMVAQASSASLTV**N**WSLGTDYTPLTTGKTFSVGDTIVFNYGAGHTVDEVSENDYKSCTLGNSITSDSSGTTTIALTTTGPRYFICGIPGHCAAGMKLAVTVAS**N**SSNGVAGGTTTPTPFTGGGGGY**N**PTTTQAIPCAAWAVSCPLRALVATWAVVFYALALS |
| AtUC5 | I | MANLCTLVGFLVIIFFNVFAPASSASHPVEWSLGKDYSSLATGKSFAVGDTIVFNYGAGHTVDEVSESDYKSCTLGNAISSDSSGTTSIALKTPGPHYFICGIPGHCTGGMKLSVIVPAASSGGSTGDGTTDKNTPVQDGKTTPSEGKKASPSASATAVLKPLDALVVTCVVALMYTLALP |
| AtUC6 | III | MKMQAVLVILVFSGLLSVKTALAARHVIGGSQGWEQSVDFDSWSSDQSFKVGDQIVFKYSELHSVVELGSETAYKSCDLGTSVNSLSSGNDVVKLSKTGTRYFACGTVGHCEQGMKIKVNVVSSDSKSASSPSGSGSGSDSGSGSGSSSGHGLRASTGYMFVVGSLVIGLIWAY |
| AtUC7 | I | MGSTAATALLLLLLLVAVPAVFAVTFQVGDNDGWTIGVEYTSWVSEKTFRVGDTLEFKYGPSHSVAVVNKADYDGCETSRPTQSFSDGDTKIDLTKVGAIHFLCLTPGHCSLGMKLAVQVLAAVSLEPPPSPSAPSPSPSAPSPSPSAPSPSPSPGNAENLKNAASKGIMSYGKIVVTMVLMYGVLN |
| AtUC8 | I | MKKTSKIQFLFNLCIIFGVVVIRRC**N**ATTYFVGDSSGWDISSDLESWTSGKRFSPGDVLMFQYSSTHSVYEVAKDNYQNC**N**TTDAIRTFTNG**N**TTVALSKPGNRFFVCGNRLHCFAGMRLLVNVEGNGPSQAPVGSPQAATSGILQPSSKKNNPATGVASSAARFVGDSGWRGTMGIFVYFMVFAFPFIWFC |
| AtSC1 | I | MALIKNNIFFTSLLIFVTLFGVAVGGTVHKVGNTKGWTMIGGDYEAWASSRVFQVGDTLVFAYNKDYHDVTEVTHNDFEMCESSKPLRRYKTGSDSISLTKPGLQHFICGVPGHCKKGQKLQIHVLPASLGHVAVPVPGPVRSQSSSSSPSPSPLVDPPVNNAPQYQMGPTPASHSAASADFIFTFSFDLTLIDLCTFFILFFILV |
| AtSC2 | I | MALIKSNAFFTSLLILVALFGISVGGTVHKVGDSDGWTIMSVNYETWASTITFQVGDSLVFKYNKDFHDVTEVTHNDYEMCEPSKPLARYETGSDIVILTKPGLQHFICGFPGHCDMGQKLQIHVLPASLGPVAAPVPGPVRPPSSFSSPSQSPLAESPVNHAPVQYQMGPSPAPHSAASNSNVWIGLCFLPLLSLLILV |
| AtSC3 | I | MAGVFKTVTFLVLVFAAVVVFAEDYDVGDDTEWTRPMDPEFYTTWATGKTFRVGDELEFDFAAGRHDVAVVSEAAFENCEKEKPISHMTVPPVKIML**N**TTGPQYFICTVGDHCRFGQKLSITVVAAGATGGATPGAGATPAPGSTPSTGGTTPPTAGGTTTPSGSSGTTTPAGNAASSLGGATFLVAFVSAVVALF |
| AtSC4 | I | MAAIIVAALACIVVMLRLSEAAVYKVGDSAGWTTIANVDYKLWASTKTFHIGDTVLFEYNPQFHNVMRVTHPMYRSC**N**TSKPISTFTTG**N**DSITLTNHGHHFFFCGVPGHCLAGQKLDLHVLLPASSTPLSDPPTSSSSSPPSTTIPAAGVPGPSPSLAASLPSMVTAQIVAVVTLLVSLAFTNFAS |
| At1g45063 | VI | MATARMKKIFSFVIVIFTLLFGCCSATVYKVGDSDGWTAKDHLYYHWTEDKEIHVGDSLIFEYDHNLNDVTQVSGGLEYEFCDSSFPKAVYNTGHDVVTFTEPGSYYFITS**N**HTQCTSGQRLGVFVVHDPSSPSPLPLPSKIIPSRHVYKVGDSKSWGVYDSDFYY**N**WSKEKQFNVGDGLLFEYNNEVNGVYEISGDLEFLNCDPTSPIAVHKTGHDIIKLTKPGIHYFISSEPGHCGAGLKLQVVVGTTLNVPKLSPLERLTRNRLHICDRFISWGIQVPSLCLLCNALDETRQHVFFDCPFSHEVWSFFCSNARVTPPRMFKDSARWLRHPCRDKKVAFILKLAYQASVYHIWRERNIRLNS**N**KSFP |
| At3g53330 | VI | MITKKIFGFVLAITILLSCCSAKIYKVGGSRGWSGKTNSWAERKEFHVGDSLIFQYHQNVNDVTQLSDALKYESC**N**SSSPKAVYNTGHDVTFLSSMKSHVRSLHHHEARPMNGHDPLAITPSPPPPSKTHERSRPITPSPPPPSKTHEPSRPNTPPPPPPPSKTHEPSRRITPSPPPPSKILPFGKIYRVGDYGGWSVYYSYYYYKWSEGKQFHVEDTLFFQYNKELNDVREITDELEFRSCESTSTVAVYKTGHDLIKLTKPGVHYFVSLKTGLCQAGIKLRVTVQPSTEAVTFPNVPKKKLSPTVNRW |
| At4g01380 | V | MGITRQSTRFHKPSLSTIETWFSIKLVFHVGDSLIFEHNHNLNDVTQVSGALEYEFCDSSSPKAVYNPGHDISWCGSKGWSVPQESYFYYRWSEKTQFPIGDSLLFEYDNEVNDVLEISGDLEFISCYPISPVAVHMTGHDLVTLTEPGVHYFISSKTPGHCYAGLKLRVVVGPLTKAVPVPNVPTKKIELSLMDRFNRWLRTFGPQPHH |

^a^, “XXX”: Signal peptide; “XXX”: PCLD, plastocyanin-like domain; “XXX”: Putative AG site; “XXX”: Putative arabinosylation site; “**XXX**”: **N-glycosylation site**; “XXX”: GPI-anchor additional signal.
