# Supplementary material for: The Phytocyanin Gene Family in Rice (Oryza sativa L.): Genome-Wide Identification, Classification and Transcriptional Analysis
Source: PLoS One. 2011 Oct 3;6(10):e25184. doi: 10.1371/journal.pone.0025184 (PMC3184959; doi:10.1371/journal.pone.0025184)
Supplement: Table S6 — Primers used in qRT-PCR of rice PC genes. (DOC) [file pone.0025184.s009.doc]

**Table S5. Primers used in real-time PCR**

| Gene | Forward | Reverse |
| --- | --- | --- |
| *UBQ5* | ACCACTTCGACCGCCACTACT | ACCACTTCGACCGCCACTACT |
| *eEF-1α* | TTTCACTCTTGGTGTGAAGCAGAT | GACTTCCTTCACGATTTCATCGTAA |
| *18S rRNA* | CTACGTCCCTGCCCTTTGTACA | ACACTTCACCGGACCATTCAA |
| *25S rRNA* | AAGGCCGAAGAGGAGAAAGGT | CGTCCCTTAGGATCGGCTTAC |
| *OsENODL4* | cccaagaacgtgtccaactc | cttaatcaagcgccatgtga |
| *OsENODL5* | cggtccggcccgtactactt | gcggcgttgatcgactgct |
| *OsENODL8* | ggtcgaggtacttcgtcagc | aggaggcagcacagcataa |
| *OsENODL9* | gcccacaggctcagagtcgt | cggtcgagcaggtacttggtg |
| *OsENODL10* | gactcggtggtgcaggtga | ggcgtggtgaggttgaaga |
| *OsENODL12* | caactggaagcccaacacc | ccgaagcagtagccgtagc |
| *OsENODL14* | ggtgatggtagtgacggaaga | cgctgatgaagtagaagaggc |
| *OsENODL16* | cgacgagacgtacaaccactg | ggcgtacttgaaatcgaggaa |
| *OsENODL17* | cgtggtgaaggtggacaag | ctggagaagtaggcgaagc |
| *OsENODL19* | cgcctgggtcaagaagcac | gccttgtcgcagttgtcgt |
| *OsENODL21* | acggctggaacccaaacat | tagcggcgtgagtcgttga |
| *OsENODL23* | tgggacacgcagaccaacc | cctccacgccgtagatgaagt |
| *OsENODL24* | gtcgaacgccaccgcctact | ccgcatcccttcctcgcact |
| *OsUCL1* | ccgttgctgccaccttctc | gcctgctcctgtgcttcttg |
| *OsUCL4* | cagcgaccagaacaccaagat | cgaccagcaggcccatcac |
| *OsUCL5* | tacaccgttggcgacagca | ccagaccagcacgcagatta |
| *OsUCL6* | gtcgttcaaagtcggagacag | gcgatggtgcagatgaagtag |
| *OsUCL7* | gctgaagaggaagccaacg | gcgacgcactatgacaacaa |
| *OsUCL8* | ttcctttgcccagtttcca | tcgcattgcactgcctctt |
| *OsUCL9* | gcccacaggctcagagtcgt | cggtcgagcaggtacttggtg |
| *OsUCL12* | catgccgtccatctcaacc | gatacaacatccgtcactcaa |
| *OsUCL14* | gggacatcagcgccgactt | ctcgctcaacgtgtggtact |
| *OsUCL15* | cggcgatacgctattgttc | gatggtcgtcccgtccttg |
| *OsUCL16* | ccgacgactccatctacga | cctaacagcactgcctcca |
| *OsUCL17* | cgtggtgaaggtggacaag | ctggagaagtaggcgaagc |
| *OsUCL18* | gtacccgaaggagatggacg | gccgctgatgaagaagaagg |
| *OsUCL20* | caccaagcccaggcacaag | gatggttgacgcagcagacg |
| *OsUCL22* | ccatcgcagattcgcaacc | cgccgaccgtgtagtcctt |
| *OsUCL23* | cttctgcttgcctcttcca | cgagcgacccactctttgt |
| *OsUCL24* | gctcatgggacttgaggac | cgccgctggagaatacttg |
| *OsUCL27* | acaccagcaacgagaaacacc | tccgcgaagacgagacagg |
| *OsUCL29* | atcgcaatgctcctcgtca | aggcgtcgtagtcgctctt |
| *OsUCL30* | cccacctgccaggatagag | cgatggaacggaacaacaac |
| *OsUCL31* | tcgtactctgccgcctctt | ggcttccacgcactgtctc |
| *OsUCL33* | caggcgaccagatagtgtt | ggaagccacagatgaagta |
| *OsSCL1* | gttcatctcctccatccaca | ccgagcatcagcaccagca |
| *OsSCL2* | gccatcacggtctccaact | gacggaaatgctgccaataa |
| *OsSCL3* | catcgtcaagcccaagagc | gcggcgaccaacccaacat |
